# Number Attraction in Pronoun Production

**DOI:** 10.1162/opmi_a_00167

**Published:** 2024-11-01

**Authors:** Margaret Kandel, Cassidy R. Wyatt, Colin Phillips

**Affiliations:** Department of Psychology, Harvard University, Cambridge, MA, USA; Faculty of Linguistics, Philology, and Phonetics, University of Oxford, Oxford, UK; Department of Linguistics, University of Maryland, College Park, College Park, MD, USA

**Keywords:** agreement attraction, pronouns, language production, reference determination

## Abstract

Pronoun production involves at least two processes: (i) deciding to refer to a referent with a pronoun instead of a full NP and (ii) determining the pronoun’s form. In the present study, we assess whether the second of these processes occurs as a by-product of the first process—namely, does accessing the message-level representation of the referent provide access to the features required to determine pronoun form, meaning that pronouns should be robust to errors, or are pronoun features determined through an agreement operation with the antecedent, in which case they may be susceptible to agreement attraction, similar to subject–verb agreement. Prior lab experiments suggest that pronouns display number attraction at a similar rate to verbs. However, in contrast to verb attraction errors, there is no documentation of systematic pronoun attraction errors in corpora of natural speech. Our study builds upon prior lab work by eliciting pronoun sentences using a scene description paradigm that engages the pronominalization processes involved in natural speech. Across three experiments, we observed small but reliable number attraction effects for pronouns, suggesting that pronoun form is not always determined from the message-level representation of the referent. The elicited error rates were smaller than those previously observed for verbs in a similar scene-description paradigm; this smaller error rate helps to reconcile the apparent discrepancy between pronoun number attraction error rates observed in and outside the lab. The results suggest that pronoun form is determined (at least at times) through an agreement process referencing the features of the linguistic antecedent.

## INTRODUCTION

The use of referential expressions such as pronouns presents different challenges for speakers than for comprehenders. Upon encountering a pronoun, comprehenders must identify the intended referent in order to interpret the speaker’s message; this process can present difficulty in cases of ambiguity when there is more than one possible pronoun referent. In contrast, this challenge is removed for speakers, who already know the message they are intending to convey. For the speaker, pronoun use involves at least two processes: (i) deciding to refer to a referent using a pronoun (reference form selection), and (ii) determining the form of the pronoun to use.

While there has been much research exploring the first of these processes—i.e., deciding whether to refer to a referent using a pronoun instead of a full NP (see Arnold & Zerkle, 2019 for review)—there has been less research investigating how a suitable pronoun form is determined once a speaker decides to pronominalize. In fact, most proposed models of reference production do not expressly include mechanisms for selecting pronoun form (Arnold & Zerkle, 2019; but cf. Schmitt et al., 1999), despite the fact that pronoun forms vary within languages and the selected form must agree with the features of the pronoun’s antecedent (e.g., number, gender). Reference production models often contain an implicit assumption that the speaker arrives at the correct pronoun form as a by-product of selecting the reference form, or deciding to use a pronoun. This assumption could be cashed out if accessing the message-level representation of a referent during the process of reference form selection provides the speaker with all of the features necessary to determine pronoun form—either because the features are salient in the message-level representation itself (e.g., notional number, conceptual gender) (Meyer & Bock, 1999) or because this representation directly activates a corresponding lemma, which in turn provides the features (e.g., Schmitt et al., 1999). Such a mechanism predicts that pronoun form selection should be robust to interference from the features of nearby nouns in the pronoun’s sentence context. If features are accessed directly via the message-level representation, the speaker should arrive at the correct pronoun form as long as they start from the correct representation. We thus should not expect to see the same systematic interference from nearby linguistic material that has been observed for other dependencies, such as subject–verb agreement. Indeed, whereas natural speech shows systematic agreement attraction errors for verbs, in which the verb erroneously agrees with the features of a noun other than the subject (e.g., *the key to the cabinets are on the table; Bock & Miller, 1991; Haskell et al., 2010; Pfau, 2009), to our knowledge, there is no comparable documentation of frequent, systematic pronoun attraction errors in the wild.^1^

However, there is evidence that pronoun attraction errors can be elicited in the lab, sometimes at comparable rates to verb attraction errors (Bock et al., 1999, 2004, 2006). These high rates of pronoun errors are potentially surprising, given that the speaker should have a clear idea of what the referent is and what its features are—especially in a language like English, in which the relevant features for determining pronoun form (number, conceptual gender) are typically salient in the conceptual representation of the referent. In addition, pronoun number serves an important communicative role: Listeners use it to identify referents. Thus, the stakes for correct pronoun agreement are high, meaning that, in an ideal system, errors should be rare. The presence of reliable, robust pronoun number errors therefore cannot be dismissed as inconsequential.

This pattern of errors has been explained by proposing common mechanisms for both verb and pronoun agreement. For instance, it has been suggested that verbs and pronouns both undergo a feature matching process that references the reconciled number of their agreement controller (an extension of the marking-and-morphing hypothesis; Eberhard et al., 2005; more below), with errors more likely to arise when the reconciled number is less clear to the speaker (e.g., due to differences in grammatical and notional number values and the influence of the number features of morphemes bound to the controller phrase). Similar attraction rates can thus arise for pronouns and verbs if they reference a common representation of the agreement controller during this feature matching process.^2^ Given that verbs display comparable rates of attraction both in and outside of the lab (e.g., Bock & Miller, 1991; Haskell et al., 2010), such an account may predict similar rates of verb and pronoun agreement attraction in natural speech (assuming the conditions for attraction arise at similar rates for both agreement targets). We thus see a discrepancy whereby models of reference form selection under-predict the errors we see in production experiments and models of pronoun agreement attraction appear to over-predict the errors we see in natural speech.

Although it is possible that this discrepancy arises because pronouns appear in attraction-inducing contexts less often in natural speech than in carefully designed elicitation tasks, another potential source of this contrast is the lab elicitation paradigms themselves. Most experiments investigating pronoun attraction have used preamble completion tasks that (i) provide the speaker with the meaning to convey in the elicited sentences via a linguistic prompt and (ii) remove the decision to pronominalize by instructing the speaker to produce a pronoun in a specified sentence construction (e.g., Bock et al., 1999, 2004, 2006; Meyer & Bock, 1999; Slevc et al., 2007). Since the speakers in these tasks do not themselves construct the message to be conveyed and use it to guide sentence formulation, instead interpreting the message from a prompt and following a sentence completion formula, it is not obvious that the speakers access the message-level representation of the referent during pronoun planning in the same way they would in natural speech. In fact, prior research has shown that using an elicitation paradigm that requires speakers to generate and describe a message de novo can change the shape of attraction effects (e.g., the markedness asymmetry for verb attraction; Kandel et al., 2022) and can even extinguish effects that appear in preamble completion tasks (e.g., attraction effects for reflexive pronouns; Kandel & Phillips, 2022).

In the present study, we investigate the conditions under which pronoun number attraction effects arise in experimental contexts in order to address the apparent discrepancy between existing proposals of pronoun form determination and previously-observed behavioral patterns. In particular, we investigate (i) whether speakers still produce pronoun agreement attraction errors in tasks that involve meaning-to-form mapping from a speaker-generated message, (ii) whether errors occur when the attractor is not within the same syntactic phrase as the antecedent (under some accounts, this should have a substantial impact on attraction error rates; e.g., Eberhard et al., 2005), and (iii) whether attraction effects persist when speakers engage the process of reference form selection in addition to that of pronoun form determination, similar to natural speech. Our investigation focuses on number agreement in particular, as number features are likely to be salient in the message-level representation of the referent that is presumably accessed during the decision to pronominalize, making number a good candidate for a feature to be provided directly from the process of referent access. We use a scene description paradigm that has been demonstrated to elicit robust number attraction effects for verbs (Kandel & Phillips, 2022; Kandel et al., 2022; see also Nozari & Omaki, 2022; Veenstra et al., 2014 for similar tasks). The presence or absence of number attraction effects for pronouns provides insight into the processes that underlie pronoun production and how pronoun form is determined, thereby enabling psycholinguistics to develop more comprehensive models of reference production. In addition, differences in the relative susceptibility of different phenomena to attraction are informative about the degree of specialization in dependency formation mechanisms.

If we find reliable and robust pronoun number attraction errors across all contexts, that would suggest that the features necessary to determine pronoun form do not come for free from the message-level representation of the referent during reference form selection, challenging the assumption made by many models of reference production. If the features were derived directly from this representation itself (e.g., Meyer & Bock’s, 1999 conceptual hypothesis) or via a direct link from the representation to lemma (e.g., Schmitt et al., 1999), there would be no opportunity for the features of nearby nouns to interfere. If the pronoun error rates resemble those elicited for verbs using the same paradigm in prior work, that could suggest that similar mechanisms or representations underlie both subject–verb and antecedent–pronoun agreement. The contexts in which errors arise are informative about what these mechanisms or representations could be. Alternatively, a lack of pronoun attraction effects would suggest that prior lab results are a consequence of the elicitation tasks used and would support models of pronoun form determination that are robust to interference. A better understanding of pronoun number attraction effects, including the rates at which they occur and the contexts in which they arise, can be used to inform the outcomes that models of pronoun form determination must be able to predict.

### Pronoun Number Attraction

Agreement attraction effects are primarily investigated in subject–verb agreement relations and occur when a nearby noun (the “attractor”) disrupts normal agreement processes, as in (1–2). Note that the attractor need not be adjacent or even the closest noun to the verb (2).

(1)  *The key to the cabinets **are** on the table (Bock & Miller, 1991)
(2)  *The cabinets that the key **open** are on the second floor (Staub, 2010)

In these sentences, the bolded verb erroneously agrees with the plural attractor noun *cabinets* instead of the singular subject *key*. In production, number attraction effects lead speakers to produce more verb number agreement errors when the agreement controller and the attractor have different number features (“mismatch” conditions) than when they have the same feature (“match” conditions); this effect is robust and reliable across many languages (e.g., Bock & Cutting, 1992; Bock & Eberhard, 1993; Bock & Miller, 1991; Den Dikken, 2001; Francis, 1986; Franck et al., 2002; Hartsuiker et al., 2003; Haskell et al., 2010; Pfau, 2009; Slioussar, 2018; Vigliocco et al., 1996; though see Chromý, Lacina, & Dotlačil, 2023 for evidence that number attraction errors are virtually non-existent in Czech). In comprehension, number attraction errors are subject to grammatical illusion and facilitation effects (e.g., Clifton et al., 1999; Dillon et al., 2013; Kaan, 2002; Lago et al., 2015; Pearlmutter et al., 1999; Shen et al., 2013; Tanner et al., 2014; Wagers et al., 2009; though see Chromý, Brand, et al., 2023; Chromý, Lacina, & Dotlačil, 2023 for evidence that agreement attraction comprehension effects are negligible or absent in Czech).

In a series of studies comparing verb and pronoun production, Bock and colleagues observed that reflexive and tag pronouns also display number attraction errors (e.g., 3–4) (Bock et al., 1999, 2004, 2006). Pronouns have additionally been found to display grammatical gender attraction (Meyer & Bock, 1999).

(3)  *The actor_i_ in the soap operas watched themselves_i_ (Bock et al., 1999)
(4)  *The actor_i_ in the soap operas rehearsed, didn’t they_i_? (Bock et al., 1999)

The number error rate profiles for pronouns are remarkably similar to those observed for verbs (Table 1), even displaying the same markedness asymmetry, with greater attraction from plural attractors than singular ones. These results led the researchers to propose that verbs and pronouns share a common mechanism for number determination—namely, both dependencies involve referencing a reconciled representation of the agreement controller’s number value that is less clear to the speaker in the mismatch cases (Eberhard et al., 2005). This proposal derives from marking-and-morphing accounts of verb attraction, which claim that the reconciled plurality of the agreement controller (e.g., a subject phrase) is a continuous property that depends upon the number of the nouns it contains in addition to notional factors such as collectivity (Eberhard et al., 2005). Under this account, agreement errors occur when the reconciled number value for the agreement controller is more ambiguous, or less clear, such as when the subject head and the attractor noun differ in number (as is the case for the subject phrase *the key to the cabinets*). Eberhard et al. (2005) extended this proposal to pronoun agreement, proposing that pronoun number is similarly influenced by the reconciled agreement controller number, in addition to the referent’s notional number.

**Table 1.** Number error rates from prior studies by Bock and colleagues.

Source	Condition	Verb	Tag Pronoun	Reflexive
Bock et al. (1999)	SS	2%	4%	2%
	SP	10%	18%	17%
	PP	1%	1%	1%
	PS	1%	2%	4%
Bock et al. (2006), Exp. 1 (American English speakers)	SS	1%	3%	2%
	SP	16%	18%	20%
	PP	2%	1%	2%
	PS	1%	2%	5%
Bock et al. (2006), Exp. 1 (British English speakers)	SS	1%	2%	1%
	SP	9%	14%	15%
	PP	1%	0%	1%
	PS	1%	2%	3%
Bock et al. (2004), Exp. 1	SS	2%	2%	
	SP	15%	18%	
	PP	3%	8%	
	PS	2%	12%	
Bock et al. (2004), Exp. 2	SS	0%	0%	
	SP	23%	17%	
Bock et al. (2004), Exp. 3	SS	2%	5%	
	SP	18%	26%	

*Note*. The condition labels describe the number (S = singular, P = plural) of the agreement controller (the subject head) and the attractor (e.g., SS = singular controller + singular attractor). In experiments with conditions containing both collective and individual subject heads or attractors, we report results from the individual head/attractor conditions only. Bock et al. (2004) only investigated verbs and tag pronouns; Experiments 2 and 3 only tested the SS and SP conditions. Rates for Bock et al. (2004) were calculated from the responses categorized by the authors as containing an unambiguously singular or plural agreement target and no preamble repetition or other form errors. In all experiments, the attractor intervened between the agreement controller (the subject head) and the agreement target. Example verb sentence (preamble underlined): *The actor in the soap opera was popular*. Example tag pronoun sentence (preamble underlined): *The actor* *in the soap opera rehearsed, didn’t he?*. Example reflexive sentence (preamble underlined): *The actor* *in the soap opera watched himself*.

The pronoun number attraction effects observed by Bock and colleagues were elicited using a preamble completion paradigm (Bock et al., 1999, 2004, 2006). In this paradigm, participants hear a short sentence fragment (a “preamble”) that they are required to repeat and complete as a full sentence. The verb-eliciting preambles consisted of a complex subject phrase containing both the agreement controller (subject head) and a potential attractor noun (e.g., *the actor in the soap operas*) that participants were instructed to repeat and complete using a verb of their choice. The pronoun-eliciting preambles consisted of a complex subject phrase followed by a verb (e.g., *the actor in the soap operas rehearsed, the actor in soap operas watched*) that participants were instructed to complete using either a tag construction (*didn’t he*/*they*) or a reflexive pronoun (*himself*/*themselves*). While variations of this paradigm are very common in the verb attraction literature (e.g., Barker et al., 2001; Bock & Cutting, 1992; Bock & Eberhard, 1993; Bock & Miller, 1991; Brehm & Bock, 2013; Franck et al., 2002; Hartsuiker & Barkhuysen, 2006; Hartsuiker et al., 2001; Haskell & MacDonald, 2003; Thornton & MacDonald, 2003; Staub, 2009, 2010; Vigliocco et al., 1996; Vigliocco & Nicol, 1998; inter alia), the task differs from natural speech production in crucial ways that have the potential to significantly alter the process of pronoun planning.

First, although this paradigm is used to study production processes, the task relies heavily on language comprehension processes, as participants must interpret the provided preamble to determine the message of the sentence to utter. For pronoun sentences, the message that speakers convey is entirely provided by the preamble together with the experiment instructions (this issue is somewhat ameliorated in the verb sentences, when speakers have more flexibility in the sentence completions). Since speakers do not generate the message by themselves, they may have weaker access to the message-level representations of the sentences they produce, which could change how pronouns are planned (though note that even if speakers do have a clear representation of the message, mapping from this message to form likely proceeds differently than in natural speech; more below). Furthermore, given that participants’ responses require interpretation of a preamble, it is possible that errors may in part reflect misinterpretations of the preamble (e.g., Ryskin et al., 2021).

Second, the preamble completion paradigm additionally involves memory processes, as participants must hold the preamble in memory as they interpret it in order to later recall and successfully repeat it during their responses (though half of the trials in Bock et al., 2006 allowed participants to read the preamble as they provided their response instead of recalling it from memory). It is possible that these memory demands may affect the likelihood and/or distribution of number errors relative to natural speech, in which it is not obvious that a speaker must hold on to so much of the prior sentence form in order to decide to pronominalize and to produce an appropriate pronoun form.

Third, whereas in natural speech, the form of an utterance is typically driven by the message, in preamble completion tasks, much of the sentence form is pre-constructed and provided in the preamble. In the case of the pronoun sentences, these preambles consist of all but the final 1–2 words of the sentence. Under these task constraints, which reduce sentence planning to what is essentially a fill-in-the-blank task, it is not obvious that the mapping from message to sentence proceeds in the same fashion that it would in more natural speech. Relatedly, in the pronoun sentences, participants do not make a decision to pronominalize in order to abide by sentence formulation constraints. Rather, they produce pronouns in order to satisfy the task instructions, which require them to complete preambles following a prescribed formula. Participants are explicitly told to produce either a sentence tag (which requires a pronoun by nature of the construction) or to complete the sentence using a reflexive pronoun. Since speakers do not make the decision to pronominalize the same way they would in natural speech, they may not access the message-level representation of the referent in the same way (especially if they have a weaker representation of the message) and may rely on task-specific strategies to determine pronoun form, such as referring to the linguistic form of the antecedent provided in the sentence fragment.

Thus, generating a pronoun in these tasks involves neither the representations nor the decisions that generally go into generating a pronoun. Given these concerns, Kandel and Phillips (2022) investigated whether number attraction effects for reflexive pronouns persist in a different task that entails more of the processes involved in natural speech. They elicited sentences using a scene description paradigm, in which speakers described the events of short scenes (see Nozari & Omaki, 2022; Veenstra et al., 2014 for additional examples of description tasks). To complete the scene description task, speakers must construct a message to convey based on the events of the scene and then map from this speaker-generated message to a sentence. The task thus avoids engaging language comprehension or explicit memory demands and also avoids providing participants with a pre-constructed sentence fragment. Furthermore, speakers are unlikely to be confused over the agreement controller identity or its features because they are visible as speakers plan the message and produce their response, meaning errors are unlikely to arise due to confusion or misinterpretation of the message intended to be elicited. In Kandel and Phillips’ (2022) experiments, speakers described scenes of aliens (*blueys*, *greenies*, *pinkies*) performing an action called *mimming*. Across experiments, Kandel and Phillips (2022) manipulated whether this action was intransitive (eliciting sentences such as *the bluey above the greeny is mimming*; Experiment 1) or reflexive (e.g., *the bluey_i_ above the greeny mimmed itself_i_*; Experiment 2). Kandel and Phillips’ (2022) Experiment 2 additionally contained filler sentences in which the action was transitive; the transitive scenes prompted speakers to produce simple object pronouns (*it*, *them*) in order to avoid repeated reference within the sentence (e.g., *the bluey above the greeny_i_ mimmed it_i_*).

This scene description paradigm elicited robust verb attraction effects (see Kandel et al., 2022 for a web-based replication) but virtually no reflexive number errors (Table 2). Crucially, when the same sentences were elicited using a preamble paradigm in the style of Bock and colleagues’ experiments (specifically modeled after the task/instructions from Bock et al., 1999), Kandel and Phillips (2022) observed number attraction errors for both verbs and reflexive pronouns. These results thus demonstrate that the choice of elicitation paradigm can influence the way that reflexive pronouns are planned and produced, suggesting that the reflexive pronoun attraction effects observed by Bock and colleagues (Bock et al., 1999, 2006) may be a by-product of the paradigm they used rather a consequence of the way that reflexive pronouns are planned in natural speech (elicitation paradigm also appeared to influence the size of the markedness asymmetry for verbs; see Kandel et al., 2022 for discussion).

**Table 2.** Number error rates for the different agreement targets in Kandel and Phillips’ (2022) scene description tasks.

Condition	Verbs	Reflexives	Object Pronouns
SS	0.4%	0.2%	0.4%
SP	25%	0.4%	3%
PP	3%	1%	2%
PS	21%	3%	6%

*Note*. In the verb and reflexive sentences, the attractor intervened between the agreement controller (the subject head) and the agreement target. In the object pronoun sentences, the attractor did not intervene between the agreement controller (the antecedent) and pronoun. Example verb sentence: *The bluey above the greeny is mimming*. Example reflexive sentence: *The bluey_i_ above the greeny mimmed itself*. Example object pronoun sentence: *The bluey above the greeny_i_ mimmed it_i_*.

Curiously, although the scene description paradigm produced no attraction effects for reflexive pronouns, Kandel and Phillips (2022) observed a small but statistically significant attraction effect for the simple object pronouns in their transitive filler sentences (Table 2). This effect was much smaller than those observed for tag pronouns by Bock and colleagues (Table 1) (though note that there were several differences between the tasks; we address this in the General Discussion), and it was also much smaller than the attraction effect observed for verbs using the same paradigm. These results may raise questions for the proposal that pronoun form is determined via the same feature matching procedure as subject–verb agreement, though it is important to note that the agreement controllers and the position of the attractor in the verb and object pronoun sentences were not identical (attractors in the pronoun sentences were non-intervening; see Bock & Miller, 1991; Staub, 2009, 2010 for evidence of different verb attraction profiles from intervening and non-intervening attractors). Nevertheless, this low incidence of pronoun errors aligns with the lack of obvious pronoun attraction errors in corpora of natural speech. The presence of an attraction effect (although small) could suggest that the pronoun form determination process is susceptible to systematic interference, meaning that pronoun form does not come for free during the process of accessing the message-level representation of the referent when the speaker plans the pronoun (or at least, it does not always come this way).

However, the pronoun number effect observed by Kandel and Phillips (2022) might have an alternative explanation. When speakers produced number errors, the number of the pronoun matched that of the subject head. In half of the experiment trials (i.e., the reflexive sentences), this noun was the correct antecedent for the produced anaphor. It is thus possible that speakers erroneously agreed with the attractor in the transitive sentences because they had to agree with the noun in this position so frequently. Furthermore, since this position was the sentence subject, this tendency could have been exacerbated by the subject bias in pronominalization (speakers tend to pronominalize subjects more often than non-subjects; e.g., Arnold, 2010; Fukumura & van Gompel, 2010; Kehler et al., 2008). Consequently, although suggestive, it is unclear from these results on their own whether the observed effect is a reliable product of the pronoun planning process. Establishing the reliability of this effect is important to our understanding of how pronoun form is determined and how this process compares to the formation of other long-distance dependencies, such as subject–verb agreement.

### Present Study

In the present study, we employed the scene description paradigm developed by Kandel and Phillips (2022) to investigate the pronoun number attraction effect in three experiments. We used the same novel aliens and *mimming* action in the present study as were used by Kandel and Phillips (2022) in order to allow for a more direct comparison to prior work. In particular, using these stimuli allows us to compare the attraction rates observed in the present study not only to those in Kandel and Phillips’ (2022) pronoun attraction experiment but also to verb errors elicited by using the same phonological verb with a different argument structure (Kandel & Phillips, 2022; Kandel et al., 2022).

Experiment 1 tested whether the pronoun number attraction effect observed by Kandel and Phillips (2022) replicates without the inclusion of reflexive trials. Experiment 2 tested whether the effect persists when the attractor intervenes between the pronoun and its antecedent, the antecedent of the pronoun is the sentence subject, and the attractor is not part of this subject representation (meaning the antecedent phrase’s number should be obvious to the speaker). Experiment 3 extended the findings of Experiments 1–2 by testing whether pronoun number attraction persists when speakers are not prompted to produce sentences containing pronouns on every trial and must decide for themselves when and whether to use a pronoun, more similar to natural speech.

In our study, we assessed the presence of attraction in two ways. First, we measured pronoun number errors and investigated whether such errors were more likely in the presence of an attractor whose number feature differs from that of the pronoun antecedent. This is the index of agreement attraction traditionally used in production studies with verbs. Second, we looked for evidence of attraction within the production time-course of error-free sentences. More specifically, we measured the likelihood of utterance-medial pauses prior to articulation of the pronoun, indicating the presence of interference even when errors were avoided. This analysis has been used with verbs to assess whether the same process and pressures that lead to errors are active on more than just the trials in which overt errors are produced (e.g., Kandel & Phillips, 2022; see Kandel et al., 2022 for discussion of how to interpret attraction timing effects). By measuring indexes of attraction in both the presence and absence of overt errors, we are able to assess how often the process that leads to pronoun number attraction is active.

## EXPERIMENT 1

Experiment 1 followed the same format as Kandel and Phillips’ (2022) Experiment 2 but only included transitive scenes (i.e., omitting the reflexive trials). This experiment tested whether the pronoun number attraction effect observed by Kandel and Phillips (2022) was a by-product of the fact that in half of the trials (the reflexive trials), the noun that served as the attractor in the pronoun trials (the subject) was the correct agreement controller. Experiment 1 further extends the results of Kandel and Phillips (2022) by looking for evidence of attraction in the articulation of correctly produced sentences. Kandel and Phillips (2022) used this analysis to assess the presence of attraction pressure for subject–verb and antecedent–reflexive agreement in the absence of overt errors, but they were unable to perform the comparable analysis for their pronoun trials due to methodological constraints.

It is important to note that in the sentences elicited in Experiment 1, the attractor does not intervene between the antecedent and the pronoun. This is a large difference from the tag pronoun experiments conducted by Bock and colleagues (Table 1), in which the attractor appeared between the pronoun and its antecedent. While non-intervening attractors have been found to induce verb agreement errors at comparable (Staub, 2009, 2010) or even slightly higher (Bock & Miller, 1991) rates than intervening attractors (see also Wagers et al., 2009 for evidence of robust comprehension effects from non-intervening attractors), there is evidence that non-intervening attraction for subject–verb agreement may result in different attraction profiles, as reflected in different sensitivity to attractor animacy and differences in timing effects (Bock & Miller, 1991; Staub, 2009, 2010). These differences have at times been attributed to different underlying processes (Bock & Miller, 1991; Staub, 2009, 2010), while others have treated them as fundamentally the same. It is possible that intervening and non-intervening attractors may also affect pronoun formulation differently; we test whether pronouns are influenced by intervening attractors in Experiments 2–3. With regards to the broader research question at hand, errors induced by either intervening or non-intervening attractors would provide evidence that pronoun formulation is susceptible to interference from representations other than that of the antecedent, suggesting that pronoun form does not come for free as part of the decision to pronominalize.

### Methods

#### Participants.

The participants in Experiment 1 were 33 native English speakers (10 F, 23 M, *M*_age_ = 35.5 years, *SD* = 11.6, range = 21–67) recruited from Amazon Mechanical Turk. An additional 12 participants were run in the task but omitted from analysis because their recordings were not saved on the data collection server or submitted by the participant via e-mail (4), because over 1/3 of their trials were omitted (see Data Processing for trial omission criteria) (7), or due to vulgar language preventing transcription of their responses (1). Participants typically completed the experiment in 20–30 minutes and were given monetary compensation ($4.00). No participant in the present study took part in more than one experiment.

#### Materials.

**Figure 1.**
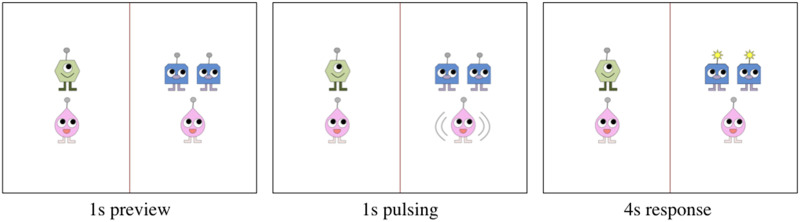
Example Experiment 2 scene eliciting the target sentence *the pinky below the blueys mimmed them*. Pulsing is indicated with grey lines.

Experimental materials consisted of short, animated scenes designed to elicit target sentences containing object pronouns. These scenes were designed following the format used by Kandel and Phillips (2022) and elicited the target sentences from the pronoun trials in Kandel and Phillips’ (2022) Experiment 2. The scenes depicted aliens (blueys, greenies, and pinkies) performing a made-up action called *mimming* (Figure 1): When one alien mims another, it pulses and then the other alien’s antenna lights up. Each scene contained two groups of aliens separated by a centered vertical line. Each group comprised two types of aliens, with one or two aliens of each type. After 1 s of preview time, the mimming action occurred in one of the alien groups: The alien(s) of one type pulsed for 1 s, and then the antenna(e) of the alien(s) of the other type within the group lit up. The antenna(e) remained lit for 4 s, after which the scene ended. Audio recording started and stopped automatically at the onset and offset of each scene.

Participants were trained to describe the actions in the scenes using sentences in the form *the* + *N*1 + *preposition* + *the* + *N*2 + *mimmed* + *pronoun*, where N1 was the subject head (and potential attractor) and N2 was the pronoun antecedent (e.g., *the pinky below the blueys_i_ mimmed them_i_*). The presence of a second alien group in each scene motivated the use of the prepositional phrase to disambiguate the sentence subject (as opposed to simply saying, e.g., *the pinky mimmed the blueys*). We manipulated the number of N1 and N2 to create four experimental conditions (Table 3). There were 24 target sentences per condition (96 total). To assess attraction, we were interested in whether there would be more pronoun form errors in the mismatch conditions (PS, SP), where the pronoun antecedent (N2) and potential attractor (N1) have different number, than the match conditions (SS, PP), where they have the same number. The relevant pairwise condition comparisons are the SS–PS conditions and the PP–SP conditions, i.e., the pairs where the pronoun antecedent (N2) has the same number.

**Table 3.** Experiment 1 conditions.

Condition	Match Condition	Example
SS	match	The pinky below the bluey mimmed it
PS	mismatch	The pinkies below the bluey mimmed it
PP	match	The pinkies below the blueys mimmed them
SP	mismatch	The pinky below the blueys mimmed them

*Note*. The condition labels describe the number (S = singular, P = plural) of the first and second noun in the sentences. In Experiment 1, the first noun was the attractor, and the second noun was the antecedent. Thus, the relevant comparisons are SS–PS and PP–SP, when the antecedent has the same number.

The presentation list and scenes were adapted from one of the presentation lists in Kandel and Phillips’s (2022) Experiment 1. The target sentences were elicited in a pseudorandomized order, and the location of the action (on the left or right side) in each scene was counterbalanced (see Kandel & Phillips, 2022 for constraints). The target group in each scene (the one in which mimming occurred) was pseudorandomly paired with another group of aliens (see Kandel & Phillips, 2022 for constraints). A subset of eight target sentences were elicited during the experiment’s practice sessions; the configurations of the scenes eliciting these sentences differed in the practice and the experimental trials.

#### Procedure.

All experiments in the present study were run using the PennController for IBEX (PCIbex; Zehr & Schwarz, 2018). Participants were asked to complete the experiments on a computer, and we recommended that they complete the experiment using the Google Chrome web browser. Participant responses were collected through the microphone connected to their computer.

We designed the experiment following the procedure described in Kandel et al. (2022, Experiment 2) to facilitate quality data collection in a web-based setting. At the start of the experiment, participants completed a recording check. During this check, participants recorded a sample response and played it back to themselves (they could repeat this process as many times as desired), allowing them to assess whether their recordings were clear and free from background noise and to make adjustments as necessary.

Participants then saw an introductory sequence (with auditory and visual instructions) to familiarize them with the task. This sequence was adapted from Kandel and Phillips (2022). Participants were introduced to the three types of aliens and the mimming action. They then practiced a simple version of the task, describing two scenes with only one group of aliens each (e.g., *the pinkies mimmed the bluey*). Participants were then shown a scene with two groups of aliens. The instructions explained that in this context, a response in the form *the pinkies mimmed the bluey* does not provide enough information to identify which alien performed the action (because there is more than one group of pinkies on screen). Participants were told that they could make their descriptions more precise using words like *above*, *below*, *to the right of*, and *to the left of*. Participants were presented with examples of these different spatial relations and practiced describing aliens by referring to their locations (e.g., *the greeny above the pinky*). The experiment instructions never explicitly told participants to use a pronoun in their responses; rather, the use of pronouns in the example descriptions in the introductory sequence and the first practice session guided participants to use pronouns in their own responses.

At the end of the introduction, participants completed two practice sessions (4 trials each). The first practice session was untimed to familiarize the participants with the task. Participants pressed a button on their keyboard to start each trial. After providing a response, they pressed a button to see the target sentence below the target group of aliens; participants used the keyboard again to end the trial. The trials in the second practice session followed the same format as the experimental trials, ending automatically 4 s after the mimming action. This time limit was intended to encourage participants to speak quickly, thereby increasing the likelihood of production errors; the response time window was 1 s longer than that used in Kandel and Phillips (2022) to accommodate the online setting (see Kandel et al., 2022). Participants were not shown the target sentences during the second practice. After completing the practice sessions, participants were presented with the 96 experimental trials.

As in Kandel et al. (2022), participants’ recordings were uploaded to a data collection server at the end of the experiment. If this upload failed, participants were able to download a .zip archive of the recordings to submit via e-mail.

#### Data Processing.

Participant responses to each trial were transcribed and coded for their inclusion of a pronoun number error. Number errors included both unrevised errors (e.g., “the pinky below the blueys mimmed it”) as well as revised errors (e.g., “the pinky below the blueys mimmed it them”). Incomplete productions of a number error were considered number errors with revision (e.g., “the pinky below the blueys mimmed i- them”).

We also noted other errors in the responses. We used the same error criteria for all experiments. Responses were omitted from the analysis if the response did not follow the target sentence formula, if the NP number marking was unidentifiable, if the response used an unidentifiable or non-standard pronoun (e.g., “thit”), if the response used a different verb (e.g., “moved”, “pinged”)^3^, or if the response expressed a meaning that did not match the action in the scene, excluding pronoun number errors (e.g., due to incorrect number marking on one or more of the NPs, use of an incorrect preposition, using the wrong alien name, etc.). If the participant corrected an error that would lead to trial omission in a single revision, the response was not omitted and was coded as containing a disfluency error. Disfluency errors additionally included false starts to a word or other word revisions (excluding agreement error revisions) (e.g., changing “him” to “it”), repeating a word or the beginning of a word, omitting a determiner, mispronunciations of a word (e.g., “thimmed” for “mimmed”), using an incomplete preposition (e.g., “the greeny right of them”), or saying the color of the alien instead of its name (e.g., “the greens”). Alternative pronunciations of the verb “mimmed” (e.g., “meemed”), using a different verb tense (e.g., “meems”), and using a non-target preposition, pronoun, or determiner with the same meaning as the target (e.g., “beneath” instead of “below”, “him”/”her” instead of “it”, “’em” instead of “them”,^4^ “a” instead of “the”) were not considered errors.

Responses containing no errors (pronoun number or disfluency) were forced-aligned to their transcriptions using the Montreal Forced Aligner v1.0.0 (McAuliffe et al., 2017) to identify the onset and offset of each word in the sentence. Sentences with a non-zero difference between the offset of the verb and the onset of the pronoun were coded as containing a pre-pronoun gap.

#### Analysis.

We analyzed (i) whether pronoun number errors were more likely in the mismatch conditions than the match conditions (the error distribution analysis) and (ii) whether mid-articulation pauses before the pronoun (gaps) in error-free sentences were more likely in the mismatch conditions than the match conditions (the pronoun timing analysis). All statistical analyses in the paper were performed in R v4.1.0 (R Core Team, 2021).

We analyzed the number error and timing data using Bayesian generalized linear mixed effects models. Bayesian estimation is able to overcome the difficulties faced by frequentist methods (such as maximum likelihood estimation) when estimating complex model structures with datasets that contain few or no observations of an outcome (e.g., a number error) in one or more cells of the analysis, as may occur in agreement attraction attraction experiments, where errors in match conditions are not common. The analysis models were fit with {rstanarm} v2.21.1 (Goodrich et al., 2020) using a Markov Chain Monte Carlo (MCMC) approach with four sampling chains of 10,000 iterations each (5,000 iterations in each chain were used for warm-up/burn-in, leaving 20,000 sampling iterations in the analysis). For each parameter, the model estimates a posterior distribution of probable values. We report the posterior medians and 95% credible intervals (CrIs) for the parameter coefficients (highest density intervals were used as CrIs). A 95% CrI comprises a range of values in which the coefficient value is 95% likely to fall; if zero is not within this range, we can be 95% confident that the parameter had a non-zero effect.

For the error distribution analysis, we constructed a generalized linear mixed model with a binomial distribution and logit link to analyze the likelihood of producing a pronoun number error. The model contained fixed effects of match (SS/PP vs. PS/SP) and antecedent number (singular vs. plural) with an interaction. The model had random intercepts for item and participant as well as a random slope of match by participant. The model was fit using a Student-*t* prior centered at 0 with 7 degrees of freedom and a scale of 2.5 for the regression coefficients and the intercept (e.g., Gelman et al., 2008). For all other priors, we used the defaults from the {rstanarm} package. For all experiments in the present study, we analyzed both the full set of non-omitted responses as well as a more restricted set without disfluency errors. In all experiments, the pattern of findings was the same in both datasets, so we report only the results from the full set of responses, which included a greater number of observations.

For the pronoun timing analysis, we constructed a generalized linear mixed model with a binomial distribution and logit link to analyze the likelihood of producing a pre-pronoun gap. The gap likelihood model used the same effects structure and priors as the error distribution analysis. We looked for localized slow-downs prior to the pronoun, as potential differences between the match and mismatch conditions earlier in the sentence cannot be confidently interpreted as pronoun planning effects. For example, in the Experiment 1 sentence frame, slowdowns directly preceding or during articulation of the verb could index verb attraction effects, slowdowns in subject phrase articulation could reflect differences in word length between match and mismatch conditions (especially in the SS vs. PS comparison), and speech onset slowdowns could reflect difficulty planning subject phrases that involve two distinct number features (Staub, 2009). Furthermore, looking for localized signatures of attraction slow-downs allows us to more easily compare timing effects across the different target sentence frames used in our study.

In our results, we report the model parameter estimates on the analysis scale (log odds). To assist with interpretation, we additionally provide probability estimates. Posterior probability distribution plots, effect plots, and fixed probability of direction (PD), effective sample size (ESS) and R-had values for our analyses are available in the Supplementary Materials. The models were computed using effects coding for the categorical fixed effects (e.g., Hardy, 1993), allowing the variables to be compared to the grand mean, analogous to a main effect in the presence of an interaction (reported in our results as the “overall” effect of the variables).

### Results

Of the 3168 responses collected in Experiment 1, 152 responses were omitted from the analysis (5%). Of the remaining 3016 responses, 169 contained disfluency errors (6%). See Supplementary Materials for a break-down of the omission and disfluency error types by condition.

#### Error Distribution Analysis.

**Figure 2.**
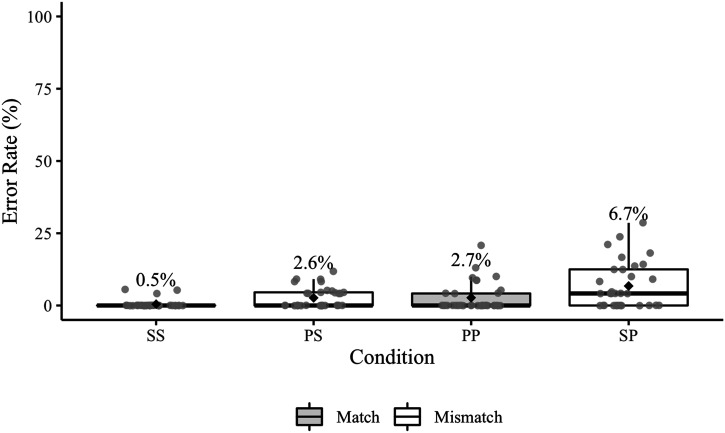
Experiment 1 participant agreement error rates. Mean error rates for each condition are labelled and identified by black diamonds. The grey points represent participant rates. The relevant condition comparisons (SS–PS, PP–SP) are positioned next to each other on the *x*-axis.

Experiment 1 elicited a total of 91 agreement errors (Table 4); 10 of these errors occurred in sentences that also contained a disfluency error. Figure 2 presents participant error rates by condition (including responses with disfluency errors). Recall that the relevant condition comparisons for Experiment 1 are SS vs. PS and PP vs. SP.

**Table 4.** Experiment 1 agreement errors and response distributions (values omitting responses with disfluency errors are given in parentheses).

Condition	Match Condition	Error Count	Response Count	Error Rate
SS	match	3 (3)	768 (733)	0.4 (0.4)%
PS	mismatch	19 (14)	749 (707)	2.5 (2.0)%
PP	match	20 (19)	758 (717)	2.6 (2.6)%
SP	mismatch	48 (45)	741 (690)	6.5 (6.5)%

In Experiment 1, the condition labels reflect the number of the attractor + the antecedent. The relevant comparisons are SS–PS and PP–SP, when the antecedent has the same number.

The posterior median for the overall effect of match was 0.99 (95% CrI [0.48, 1.57]), indicating that errors were more likely in the mismatch conditions. The posterior median for the overall effect of antecedent number was 0.81 (95% CrI [0.44, 1.23]), indicating that errors were more likely in conditions with plural antecedents. The posterior median of the interaction was −0.22 (95% CrI [−0.63, 0.15]), suggesting that there was not a reliable difference in the mismatch effect between conditions with singular and plural antecedents (see Jaccard, 2001 for discussion of how to interpret interaction coefficients in logistic regression). The estimated probabilities of agreement errors were 0.1% (95% CrI [0.0, 0.5]) in the SS condition, 0.9% (95% CrI [0.3, 2.3]) in the PS condition, 1.3% (95% CrI [0.6, 2.6]) in the PP condition, and 4.2% (95% CrI [2.2, 7.0]) in the SP condition.

#### Pronoun Timing Analysis.

**Figure 3.**
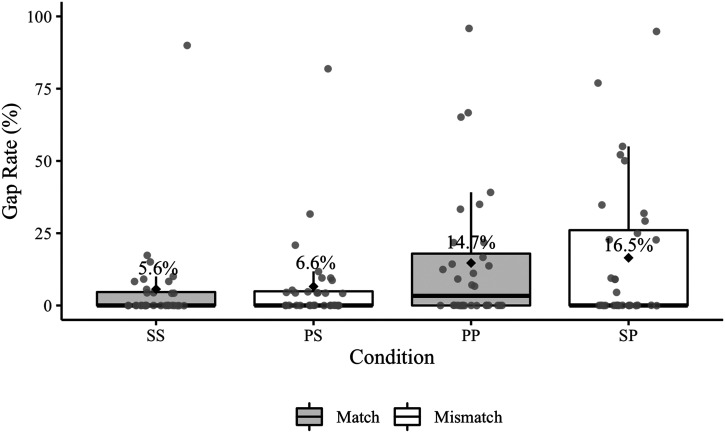
Experiment 1 participant pre-pronoun gap rates. Mean error rates for each condition are labelled and identified by black diamonds. The grey points represent participant rates. The relevant condition comparisons (SS–PS, PP–SP) are positioned next to each other on the *x*-axis.

The pronoun timing analysis was performed on 2702 responses containing no agreement or disfluency errors (one participant’s data was omitted from the analysis because their responses could not be aligned). There were 269 pre-pronoun gaps in the aligned responses (Table 5; see Figure 3 for participant gap rates). A plot showing the production time-course of the responses in the match and mismatch conditions broken down by sentence region is available in the Supplementary Materials.

**Table 5.** Experiment 1 pre-pronoun gap and response distributions.

Condition	Match Condition	Gap Count	Response Count	Gap Rate
SS	match	29	715	4.1%
PS	mismatch	36	678	5.3%
PP	match	101	678	14.9%
SP	mismatch	103	631	16.3%

In Experiment 1, the condition labels reflect the number of the attractor + the antecedent. The relevant comparisons are SS–PS and PP–SP, when the antecedent has the same number.

The posterior median for the overall effect of match was 0.16 (95% CrI [−4.40, 2.69]), indicating that pre-pronoun gaps were not reliably more likely in the mismatch conditions. The posterior median for the overall effect of antecedent number was 0.16 (95% CrI [0.69, 1.03]), indicating that gaps were more likely in sentences with a plural antecedent (i.e., gaps were more likely before *them* than *it*). The posterior median of the interaction was −0.01 (95% CrI [−0.18, 0.16]), suggesting that there was not a reliable difference in the mismatch effect between conditions with singular and plural antecedents. The estimated probabilities of pre-pronoun gaps were 1.0% (95% CrI [0.4, 2.6]) in the SS condition, 7.5% (95% CrI [3.1, 15.3]) in the PS condition, 5.7% (95% CrI [2.2, 12.4]) in the PP condition, and 1.4% (95% CrI [0.5, 3.4]) in the SP condition.

### Experiment 1 Summary

Experiment 1 replicated the pronoun number attraction error effect observed by Kandel and Phillips (2022): Pronoun number errors were more likely in the mismatch conditions than the match conditions. As in Kandel and Phillips (2022), there was no interaction between match and antecedent number, suggesting that the mismatch effect does not differ between the SS vs. PS and PP vs. SP comparisons. These results suggest that the pronoun number attraction error effect observed by Kandel and Phillips (2022) is reliable and was not a by-product of the inclusion of reflexive trials in the same experiment. In fact, in a post-hoc analysis of the data from the two experiments, there was no difference in the size of the mismatch effect in Experiment 1 and in Kandel and Phillips’ (2022) pronoun trials (match × experiment posterior median −0.01, 95% CrI [−0.25, 0.26]; see Supplementary Materials).

Nevertheless, the observed attraction effect is small, with pronoun number errors in 2% of match trials and only 4% of mismatch trials. These error rates are much smaller than those in studies using the same elicitation paradigm for verbs (1–2% errors in the match trials vs. 17–23% in the mismatch trials; Kandel et al., 2022). Moreover, these error rates are small relative to those observed by Bock and colleagues for tag pronouns with singular antecedents and non-collective attractors (0–4% errors in the SS condition vs. 14–18% in the SP condition; see Table 1), and they do not display the same markedness asymmetry (Bock et al., 1999 and Bock et al., 2006 observed little difference in error rates between conditions with plural antecedents)—we address this finding further in the General Discussion.

Given the small size of the mismatch error effect, there may be concerns about its viability as an agreement attraction effect. For example, one may be concerned that this effect is instead driven by an *it* bias, or a bias towards producing the pronoun form *it* resulting from frequency differences between *it* and *them* (e.g., in SUBTLEX-US, there are 18896.31 occurrences per million for *it* vs. 1778.82 per million for *them*; Brysbaert & New, 2009). Indeed, we observed reliable antecedent number effects in both the error distribution and pronoun timing analyses such that participants were more likely to erroneously say *it* than *them* and that pre-pronoun gaps in articulation were more likely before *them* than *it*, suggesting increased processing difficulty. However, such an *it* bias does not appear to be the cause of the observed mismatch effect. In the error distribution analysis, there was no interaction between match and antecedent number, suggesting that the mismatch effect is present independent of whether the participant is supposed to say *it* or *them*. Furthermore, if one performs the pairwise comparisons between conditions (using the same analysis model structure as the error distribution analysis with dummy-coding instead of effects coding), there are reliable differences between both the SS vs. PS conditions (posterior median 2.10, 95% CrI [0.77, 3.56]) and the PP vs. SP conditions (posterior median 1.51, 95% CrI [0.56, 2.58]). An *it* bias would not explain why erroneous uses of *it* are more likely in the SP condition than the PP condition (a simple bias towards saying *it* should produce comparable levels of errors in both conditions), nor would it explain why erroneous use of *them* is more likely in the PS condition than the SS condition (erroneous use of *them* is not predicted at all by an *it* bias). Thus, we cannot attribute the observed mismatch error effect to an *it* bias.

Relatedly, there may be concerns that apparent pronoun number errors are caused by the use of singular *them*, especially since the gender of the aliens in the scenes could be interpreted as ambiguous or neutral. However, use of singular *them* cannot explain our data pattern. Consistent use of singular *them* predicts similar rates of producing *them* in both the PS and SS conditions when the pronoun antecedent is singular; there is no reason to expect that singular antecedents are more likely to be referred to using singular *them* in one condition over the other. Thus, use of singular *them* would not produce the observed difference in the use of *them* between the PP and SP conditions. Furthermore, the use of singular *them* does not predict erroneous use of *it* in the PS condition leading to the observed difference in the SS vs. PS comparison (when the target pronoun form is *them*). Consequently, the mismatch error effect we observe cannot reduce to use of singular *them*.

The observed error effect thus appears to be an agreement attraction effect rather than reducible to other factors. This attraction effect indicates that other representations within a sentence can interfere with pronoun formulation. These results suggest that pronoun form is not always derived directly from the message-level representation of the referent, even when the relevant feature (number) is likely salient from this representation. However, it is possible that attraction errors in Experiment 1 only arose because the attractor was particularly prominent or salient, given that the attractor was the sentence subject—this possibility is addressed in Experiment 2.

Interestingly, despite observing an attraction effect in our error measure, we did not observe parallel evidence of attraction in the pronoun timing analysis. This finding differs from the overall pattern observed by Kandel and Phillips (2022) for verbs and reflexive pronouns, in which the presence or absence of an attraction error effect always corresponded to a presence or absence of a corresponding timing effect. The absence of a timing effect suggests that the process leading to attraction in Experiment 1 is infrequent, prompting errors on a small proportion of trials, with few or no additional trials on which the process was active. The pattern of results we observe resembles that for non-intervening attraction in subject–verb agreement: While there is evidence from error and response time data that intervening attractors influence verb agreement on all or most trials of an experiment (independent of whether an error was made), it has been argued that non-intervening attractors only influence a subset of trials (many of which result in overt errors) (Staub, 2010). Thus, the lack of an observable timing effect in our results could arise due the fact that the attractor in the Experiment 1 sentences (the sentence subject) did not intervene between the pronoun and the antecedent. It is possible that intervening attractors may have a larger influence on the pronoun formulation process than non-intervening attractors; indeed, in the experiments by Bock and colleagues that found pronoun number attraction rates comparable to verbs, the attractor noun always intervened between the pronoun and its antecedent (Bock et al., 1999, 2004, 2006). This is another factor that we changed in Experiment 2.

## EXPERIMENT 2

The pronoun number attraction errors observed in Experiment 1 provide evidence that pronoun form is not always determined from accessing the message-level representation of the referent, since we observed systematic interference from nearby linguistic material—i.e., the attractor noun. These results could suggest that pronouns undergo a feature matching operation with their antecedent, similar to subject–verb agreement.

As mentioned in the Introduction, Eberhard et al. (2005) propose more specifically that pronoun number attraction arises through a marking-and-morphing process, in which errors are more likely to occur when the reconciled number value of the antecedent phrase is ambiguous (e.g., when it contains multiple nouns of different number, as in *the actor in the soap operas*) and/or when there is ambiguity in the referent’s notional number (e.g., for collectives). Experiment 2 tested this hypothesis by assessing whether pronoun number attraction effects like that observed in Experiment 1 arise when there is no ambiguity in the referent’s notional number, the pronoun’s antecedent is the subject of the sentence, and the antecedent phrase only contains one noun, meaning that its marking-and-morphing number value should be unambiguous (see Supplementary Materials for the calculation of pronoun number in the Experiment 2 target sentence construction using Eberhard et al.’s (2005) marking-and-morphing approach).^5^

The Experiment 2 target sentences furthermore addressed two potential concerns from Experiment 1. Since the sentence subject is the pronoun antecedent rather than the attractor, Experiment 2 allowed us to assess whether pronoun number attraction arises from attractors that do not have the increased prominence or salience of a sentence subject (contra Experiment 1). In addition, in the Experiment 2 target sentences, the attractor noun intervened between the pronoun and its antecedent, more similar to the pronoun experiments by Bock and colleagues (Bock et al., 1999, 2004, 2006) as well as a large majority of number attraction experiments with verbs (including Kandel & Phillips, 2022; Kandel et al., 2022).

### Methods

#### Participants.

The participants in Experiment 2 were 35 native English speakers (11 F, 23 M, *M*_age_ = 35.6 years, *SD* = 11.3, range = 20–65; demographic data missing from one participant) recruited from Amazon Mechanical Turk. An additional 9 participants were run in the task but omitted from analysis because they did not complete the experiment (1), their recordings did not contain any sound (1), because they did not use consistent pronoun agreement^6^ (3), or because over 1/3 of their trials were omitted (see Experiment 1 methods for trial omission criteria) (5). Participants typically completed the experiment in 20–30 minutes and were given monetary compensation ($4.00).

#### Materials.

**Figure 4.**
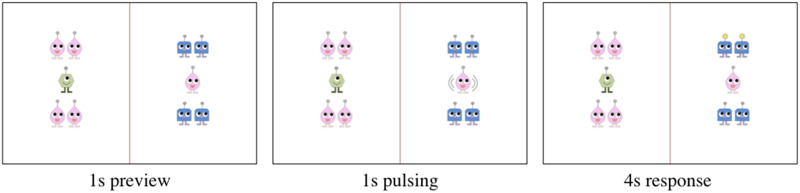
Example Experiment 2 scene eliciting the target sentence *the pinky mimmed the blueys above it*. Pulsing is indicated with grey lines.

The materials used in Experiment 2 followed the same format as Experiment 1, only the aliens were arranged in different configurations so as to elicit a target sentence structure in which the sentence subject was the pronoun antecedent. As in Experiment 1, each trial showed a scene with two groups of aliens separated by a red line (Figure 4). In each trial, the mimming action occurred in one of the two groups (the target group). One sub-group of aliens (1–2 aliens of the same type) was positioned in the center of each group (the “center set”) with two identical sub-groups (1–2 aliens of the same type) positioned around it (the “object sets”). The object sets could be positioned above, below, or to the left/right of the central set. The alien types of the center set and the object sets were always different, thus each group contained two alien types total. In the target group, the alien(s) in the central set mimmed the alien(s) in one of the two object sets.

Participants were trained to describe the actions in the scenes using sentences in the form *the* + *N*1 + *mimmed* + *the* + *N*2 + *preposition* + *pronoun*, where N1 was the subject head (and pronoun antecedent) and N2 was the potential attractor (e.g., *the pinky_i_ mimmed the blueys above it_i_*). The presence of two object sets in the target group, both containing aliens of the same type and number, provided a pragmatic reason for participants to use a higher level of detail in their descriptions in order to identify on which of the two object sets the mimming action was performed (e.g., *the blueys above the pinky* vs. *the blueys below the pinky*). The presence of two alien groups in each scene was used to prevent participants from being able to predict which alien types would correspond to N1 and N2 before the action occurred simply based on the number and alien types of the center and object sets, thereby preventing them from planning the subject and object NPs in advance of the mimming action.

We manipulated the number of N1 and N2 to create four experimental conditions (Table 6). There were 24 target sentences per condition (96 total). As in Experiment 1, to assess attraction, we were interested in whether there would be more pronoun form errors in the mismatch conditions (SP, PS), where the pronoun antecedent (N1) and attractor (N2) have different number, than the match conditions (SS, PP), where they have the same number. The relevant pairwise condition comparisons are the SS–SP conditions and the PP–PS conditions, i.e., the pairs where the pronoun antecedent (N1) has the same number.

**Table 6.** Experiment 2 conditions.

Condition	Match Condition	Example
SS	match	The pinky mimmed the bluey above it
SP	mismatch	The pinky mimmed the blueys above it
PP	match	The pinkies mimmed the blueys above them
PS	mismatch	The pinkies mimmed the bluey above them

*Note*. The condition labels describe the number (S = singular, P = plural) of the first and second noun in the sentences. In Experiment 2, the first noun was the antecedent, and the second noun was the attractor. Thus, the relevant comparisons are SS–SP and PP–PS, when the antecedent has the same number.

The presentation list (practice and experimental trials) was adapted from Experiment 1 such that the aliens corresponding to N1 and N2 appeared as the same type and in the same spatial relation in the same order (e.g., the Experiment 1 target sentence *the pinky below the blueys mimmed them* corresponded to the Experiment 2 target sentence *the pinky mimmed the blueys above it*). The side of the target group followed the same order as Experiment 1, thus the mimming action occurred an equal number of times on the left and right side of the scenes, with the mimming action occurring on the same side of the screen no more than three times in a row. The configuration of the non-target group was also adapted from the corresponding Experiment 1 scene (see Supplementary Materials for details). The aliens in the center and object sets in the non-target group differed from the aliens in the corresponding positions in the target group by at least one dimension (number and/or type) so that no set of aliens in the non-target group could be referred to by the same label (e.g., “the greenies”) as any of the sets in the target group.

In the target groups, the relative positions of the aliens corresponding to N1 (the center set) and N2 (the object set that was mimmed) was determined based on their relation in the corresponding Experiment 1 scene. To ensure variability in the positioning of the aliens in the scenes, the alien configurations in the target and non-target groups were pseudorandomized such that there were an equal number of symmetric configurations for the target and non-target groups, with no more than three symmetric target or non-target groups appearing in a row. For symmetric group configurations, the position of the object set not corresponding to N2 was positioned opposite the object set corresponding to N2. For asymmetric target group configurations, the position of the object alien set not corresponding to N2 was randomly selected from the remaining possible positions such that the group configuration was not symmetrical. For asymmetric non-target group configurations, one object set had the same pseudorandomized position with respect to the center set as the two alien types had in the non-target group of the corresponding Experiment 1 scene. The position of the other object set was pseudorandomized such that the configuration was not symmetrical.

#### Procedure.

The experiment followed the same general procedure as Experiment 1, with a few modifications made to the introductory sequence to accommodate the new target sentence format. As in Experiment 1, the introductory sequence first introduced the three types of aliens and the mimming action and then proceeded to a simple version of the task in which participants described two scenes with only one group of aliens each. Participants were then shown a group of aliens in the Experiment 2 configuration and were told that in this context, a response in the form *the pinkies mimmed the bluey* does not provide enough information to identify which alien was mimmed (i.e., because there is more than one bluey). Participants were told that they could make their descriptions more precise using words like *above*, *below*, *to the right of*, and *to the left of*. Participants were presented with examples of aliens mimming in these different spatial relations and practiced describing the scenes by referring to the locations of the mimmed aliens (e.g., *the greeny mimmed the bluey above it*). As in Experiment 1, the introductory sequence never explicitly told participants to use a pronoun in their responses, though the utility of using a pronoun was mentioned after the first practice trial (“Note that a sentence like *the pinky mimmed the greenies below it* is a better response than *the pinky mimmed the greenies below the pinky*, as it is more efficient and will help you save time in your descriptions (this will be important later on).”). While this statement introduces reduced production time as a motivation for pronominalization, pronouns were primarily motivated by virtue of being a more natural formulation that avoids NP repetition. The practice trials otherwise followed the same format as in Experiment 1.

#### Data Processing and Analyses.

The data processing procedure and analyses followed the same format as in Experiment 1.

### Results

Of the 3360 responses collected in Experiment 2, 239 responses were omitted from the analysis (7%). Of the remaining 3121 responses, 219 contained disfluency errors (7%). See Supplementary Materials for a break-down of the omission and disfluency error types by condition.

#### Error Distribution Analysis.

**Figure 5.**
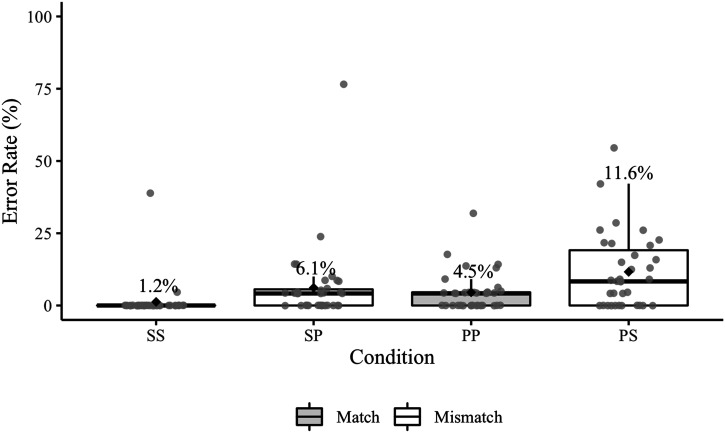
Experiment 2 participant agreement error rates. Mean error rates for each condition are labelled and identified by black diamonds. The grey points represent participant rates. The relevant condition comparisons (SS–SP, PP–PS) are positioned next to each other on the *x*-axis.

Experiment 2 elicited a total of 172 agreement errors (Table 7); 26 of these errors occurred in sentences that also contained a disfluency error. Figure 5 presents participant error rates by condition (including responses with disfluency errors). Recall that the relevant condition comparisons for Experiment 2 are SS vs. SP and PP vs. PS.

**Table 7.** Experiment 2 agreement errors and response distributions (values omitting responses with disfluency errors are given in parentheses).

Condition	Match Condition	Error Count	Response Count	Error Rate
SS	match	8 (7)	807 (750)	1.0 (0.9)%
SP	mismatch	43 (35)	776 (727)	5.5 (4.8)%
PP	match	34 (30)	771 (717)	4.4 (4.2)%
PS	mismatch	87 (74)	767 (708)	11.3 (10.5)%

In Experiment 2, the condition labels reflect the number of the antecedent + the attractor. The relevant comparisons are SS–SP and PP–PS, when the antecedent has the same number.

The posterior median for the overall effect of match was 0.87 (95% CrI [0.50, 1.28]), indicating that errors were more likely in the mismatch conditions. The posterior median for the overall effect of antecedent number was 0.65 (95% CrI [0.39, 0.93]), indicating that errors were more likely in conditions with plural antecedents. The posterior median of the interaction was −0.20 (95% CrI [−0.47, 0.06]), suggesting that there was not a reliable difference in the mismatch effect between conditions with singular and plural antecedents. The estimated probabilities of agreement errors were 0.4% (95% CrI [0.1, 0.9]) in the SS condition, 2.0% in the SP condition (95% CrI [0.8, 3.9]), 3.0% in the PP condition (95% CrI [1.5, 5.4]), and 7.0% in the PS condition (95% CrI [3.8, 11.7]).

#### Pronoun Timing Analysis.

**Figure 6.**
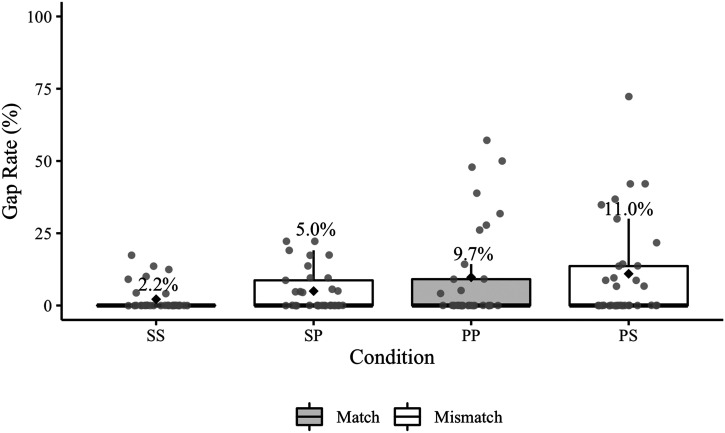
Experiment 2 participant pre-pronoun gap rates. Mean error rates for each condition are labelled and identified by black diamonds. The grey points represent participant rates. The relevant condition comparisons (SS–SP, PP–PS) are positioned next to each other on the *x*-axis.

Two participants were omitted from the pronoun timing analysis either because they had no trials that were free of disfluency errors (1 participant omitted the N1 determiner on all trials) or because the majority of their data could not be forced aligned (for 1 participant, only 2 trials could be aligned). One additional response was omitted from the timing analysis because it could not be aligned. The pronoun timing analysis was thus performed on 2663 responses containing no agreement or disfluency errors. There were 185 pre-pronoun gaps in the aligned responses (Table 8; see Figure 6 for participant gap rates). A plot showing the production time-course of the responses in the match and mismatch conditions broken down by sentence region is available in the Supplementary Materials.

**Table 8.** Experiment 2 pre-pronoun gap and response distributions.

Condition	Match Condition	Gap Count	Response Count	Gap Rate
SS	match	16	719	2.2%
SP	mismatch	34	668	5.1%
PP	match	66	665	9.9%
PS	mismatch	69	611	11.3%

In Experiment 2, the condition labels reflect the number of the antecedent + the attractor. The relevant comparisons are SS–SP and PP–PS, when the antecedent has the same number.

The posterior median for the overall effect of match was 0.67 (95% CrI [0.24, 1.18]), indicating that pre-pronoun gaps were reliably more likely in the mismatch conditions. The posterior median for the overall effect of antecedent number was 0.76 (95% CrI [0.53, 1.02]), indicating that gaps were more likely in sentences with a plural antecedent (i.e., gaps were more likely before *them* than *it*). The posterior median of the interaction was −0.24 (95% CrI [−0.48, 0.01]), suggesting that there was not a reliable difference in the mismatch effect between conditions with singular and plural antecedents. The estimated probabilities of pre-pronoun gaps were 0.3% (95% CrI [0.1, 1.0]) in the SS condition, 1.8% (95% CrI [0.8, 3.9]) in the SP condition, 2.2% (95% CrI [0.6, 5.9]) in the PP condition, and 5.1% (95% CrI [2.3, 10.0]) in the PS condition.

### Experiment 2 Summary

The distribution of pronoun number agreement errors in Experiment 2 closely resembled that in Experiment 1. Similar to Experiment 1, Experiment 2 elicited a pronoun number attraction effect such that errors were more likely in the mismatch conditions than the match conditions. As in Experiment 1, there was no reliable interaction between match and antecedent number, suggesting that the mismatch effect does not differ between the SS vs. SP and PP vs. PS comparisons^7^, though there was a reliable effect of antecedent number such that errors were more likely when the pronoun antecedent was plural (i.e., erroneous use of *it* was more common than erroneous use of *them*). These results suggest that attractor nouns do not need to be sentence subjects in order to interfere with pronoun formulation and that attraction errors arise even when the attractor is not part of the antecedent phrase and there is no ambiguity in the referent’s notional number (counter to the predictions of Eberhard et al., 2005’s marking-and-morphing model).

Nevertheless, the Experiment 2 attraction effect was still small compared to prior results with verbs and tag pronouns (see Experiment 1 Summary), with pronoun number errors in 3% of match trials and 8% of mismatch trials. A post-hoc comparison analysis suggested no reliable difference in the size of the mismatch effect in Experiments 1 and 2 (match × experiment posterior median 0.003, 95% CrI [−0.27, 0.28]; see Supplementary Materials). Thus, even though pronoun formulation can at times be susceptible to attraction interference, participants still produce the correct pronoun form on the majority of trials.

The results of the Experiment 2 pronoun timing analysis parallel the error results. Participants were more likely to pause in the same environments where pronoun number errors were more likely, even when no error was produced: Pre-pronoun gaps were more likely in the mismatch conditions than the match conditions and when the antecedent was plural (there was no reliable interaction between match and antecedent number). The fact that we observe a mismatch effect in both error and timing measures could indicate that the processes and pressures that lead to pronoun number attraction are active on more than just the cases when errors occur. However, the mismatch gap effect, like the error effect, was small, suggesting that, unlike in the case of subject–verb agreement (e.g., Kandel et al., 2022; Staub, 2010), the influence of intervening attractors on antecedent–pronoun agreement is not active on most trials.

The results of Experiments 1–2 thus provide evidence that pronoun form is at times determined through an agreement process that is susceptible to number attraction, rather than through direct access to the relevant features from the message-level representation of the referent. This result is potentially surprising, as in natural speech, this message-level referent is likely accessed during the decision to pronominalize, and the relevant feature (number) should be salient in this representation. However, it is not obvious that the participants in Experiments 1–2 were making a decision to pronominalize; although participants were never explicitly instructed to use pronouns in their responses, they repeatedly produced sentences of the same construction containing a pronoun in every trial of the experiment. Removing the decision to pronominalize may lead speakers to differently access/consult the message-level representation of the antecedent referent than they would in natural speech, potentially causing speakers to rely less on this representation when determining pronoun form, thus making them more prone to errors. Furthermore, the fact that participants produced highly similar sentences containing pronouns on every trial of the experiment could have artificially raised error rates due to interference from sentence representations across trials. We addressed these issues in Experiment 3.

## EXPERIMENT 3

Experiment 3 tested whether the pronoun number attraction effects observed in Experiments 1–2 persist when speakers produce a variety of different sentence constructions and decide for themselves when and whether to use a pronoun, more similar to natural speech. In Experiment 3, we elicited sentences in three different constructions. One of the constructions created a context in which a speaker can produce a pronoun to avoid NP repetition. This pronoun-inducing context only arose in one third of the experimental trials, and participants did not see any examples of this context in the explanation of the task. Thus, Experiment 3 allowed us to better test whether pronoun number attraction errors arise when speakers engage both the process of reference form selection and that of pronoun form determination, as is required in natural speech.

### Methods

#### Participants.

The participants in Experiment 3 were 33 native English speakers (13 F, 20 M, *M*_age_ = 34.4 years, *SD* = 10.3, range = 21–57) recruited from Amazon Mechanical Turk. An additional 19 participants were run in the task but omitted from analysis because their recordings were not saved on the experiment server or emailed to us by the participant (1), because their recordings did not contain any sound (2), because they consistently used *itself*/*themselves* instead of *it*/*them* in their responses (1), because they did not use consistent pronoun agreement^8^ (5), or because over 1/3 of the trial type included in the analysis were omitted (i.e., the pronoun trials; more below)^9^ (10). Participants typically completed the experiment in 20–30 minutes and were given monetary compensation ($4.00).

#### Materials.

**Figure 7.**
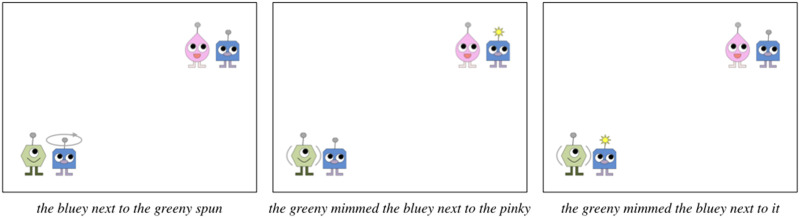
Example Experiment 3 actions. The aliens that spun or pulsed in the actions are indicated with grey lines.

As in Experiments 1–2, the experimental materials consisted of short, animated scenes with blueys, greenies, and pinkies. In Experiment 3, aliens could either mim other aliens or they could spin (Figure 7). Each scene consisted of two groups of aliens, each containing two types of aliens (with 1–2 aliens of each type). Within each group, the two sub-groups of aliens were positioned next to each other. The two groups were arranged diagonally opposed to each other. Unlike in Experiments 1–2, the groups were not separated by a vertical line. After 1 s of preview time, one sub-group of aliens on the screen (the “agent set”) performed an action. If the agent set mimmed another set of aliens, they pulsed for 1 s, and then the antenna(e) of the alien(s) in the other set lit up (this set could either be within the same group as the agent set or in the opposing group). If the agent set spun, they spun for 1.5 s. The scene remained on screen for 4 s after the action concluded. As in the prior experiments, audio recordings started and stopped automatically at the onset and offset of each scene.

The experiment scenes were designed to elicit target sentences in three different formats (Tables 9–10). The intended target sentence formats were: (i) *the* + *N*1 + *next to* + *the* + *N*2 *spun* (the intransitive filler sentences; e.g., *the bluey next to the greeny spun*), (ii) *the* + *N*1 + *mimmed* + *the* + *N*2 + *next to* + *the* + *N*3 (the transitive filler sentences; e.g., *the greeny mimmed the bluey next to the pinky*), and (iii) *the* + *N*1 + *mimmed* + *the* + *N*2 + *next to* + *pronoun*, where the pronoun indexed N1 (the pronoun sentences; e.g., *the greeny_i_ mimmed the bluey next to it_i_*). Our analyses focus on the pronoun sentences.

**Table 9.** Experiment 3 pronoun conditions.

Condition	Match Condition	Example
SS	match	The greeny mimmed the bluey next to it
SP	mismatch	The greeny mimmed the blueys next to it
PP	match	The greenies mimmed the blueys next to them
PS	mismatch	The greenies mimmed the blueys next to them

*Note*. The condition labels describe the number (S = singular, P = plural) of the first and second noun in the sentences. In Experiment 3, the first noun was the antecedent, and the second noun was the attractor. Thus, the relevant comparisons are SS–SP and PP–PS, when the antecedent has the same number.

**Table 10.** Experiment 3 filler conditions.

Type	Condition	Example
Intransitive filler	SS	The greeny next to the bluey spun
	SP	The greeny next to the blueys spun
	PP	The greenies next to the blueys spun
	PS	The greenies next to the bluey spun
Transitive filler	SSS	The greeny mimmed the bluey next to the pinky
	SSP	The greeny mimmed the bluey next to the pinkies
	SPP	The greeny mimmed the blueys next to the pinkies
	PPP	The greenies mimmed the blueys next to the pinkies
	PPS	The greenies mimmed the blueys next to the pinky
	PSS	The greenies mimmed the bluey next to the pinky

*Note*. The condition labels describe the number (S = singular, P = plural) of the first, second, and third noun in the sentences.

We manipulated N1, N2, and N3 in the target sentences to rotate through all possible combinations of alien types and number (singular, plural). There were 96 total target sentences (24 intransitive filler sentences, 48 transitive filler sentences, and 24 pronoun sentences). The target sentences were organized into two presentation lists. Both lists elicited all possible intransitive filler and pronoun sentences (24 each). The 48 transitive filler sentences were divided between the two presentation lists (24 per list) such that (i) each alien type appeared in each sentence position (N1, N2, N3) with each possible number (singular, plural) an equal number of times in each list and (ii) each list contained an equal number of each transitive filler condition (SSS, SSP, SPS, SPP, PPP, PPS, PSP, PSS). Thus, each experiment list elicited 72 target sentences (24 of each sentence type). Fourteen participants saw list 1 and 19 participants saw list 2.

The presence of two groups of aliens in each scene motivated the use of the prepositional phrase *next to* in the target sentences to disambiguate either the sentence subject (in the intransitive filler sentences) or the object of the mimming action (in the transitive filler and pronoun sentences). Each scene contained all three types of aliens. In the scenes eliciting intransitive filler sentences (the intransitive filler scenes), the group of aliens opposing the one containing the agent set always contained a set of aliens identical to the agent set (so that e.g., participants could not simply say *the bluey spun*). The aliens next to the agent set and the identical set differed in type so that participants could reference the aliens next to the agent set to disambiguate it (e.g., *the bluey next to the greeny* as opposed to *the bluey next to the pinky*; Figure 7). In the scenes eliciting transitive filler sentences (the transitive filler scenes) and pronoun sentences (the pronoun scenes), the scene always contained another set of aliens identical to the mimmed set (so that e.g., participants could not simply say *the greeny mimmed the bluey*). In the transitive filler scenes, the mimmed set of aliens was always in the group opposite the one containing the agent set. In the pronoun scenes, the mimmed set of aliens was always in the same group as the agent set. In both the transitive and pronoun scenes, the third group of aliens was always a different type from the agent set and the mimmed/identical sets. Crucially, in the pronoun sentences, in order to disambiguate which aliens were mimmed by the agent set, the participants needed to refer to the agent set in the disambiguating prepositional phrase. This necessity for repeated reference was intended to prompt participants to use a pronoun in their responses (instead of saying, e.g., *the greeny_i_ mimmed the bluey next to the greeny_i_*).

We used the pronoun sentences to assess the presence of number attraction (Table 9). As in Experiments 1–2, we were interested in whether there would be more pronoun form errors in the mismatch conditions (SP, PS), where the pronoun antecedent (N1) and attractor (N2) have different number, than the match conditions (SS, PP), where they have the same number. The relevant pairwise condition comparisons are the SS–SP conditions and the PP–PS conditions, i.e., the pairs where the pronoun antecedent (N1) has the same number.

The location of the group containing the agent set in each scene was pseudorandomly assigned such that it appeared an equal number of times in each quadrant of the screen for each sentence type/list. The position of the agent set within its group was pseudorandomly assigned such that the agent set appeared an equal number of times on the left and right side of the group for each sentence type and list. The within-group positions of the set identical to the agent set in the intransitive filler sentences, the mimmed alien set in the transitive filler sentences, and the set identical to the mimmed alien set in the pronoun sentences were similarly pseudorandomly assigned and counterbalanced. In the intransitive filler and pronoun scenes, the alien set not corresponding to N1, N2, or the set identical to N1/N2 was always a different type from the N1 and N2 aliens, and its number (singular, plural) was pseudorandomized such that for each target sentence type, the scenes contained an equal number of singular and plural aliens of each type in each condition (SS, SP, PP, PS). The same overall scene configuration did not appear more than once in either of the two experiment lists.

A subset of ten target sentences were elicited during the experiment’s practice sessions (five per session). The configurations of the scenes eliciting these sentences differed in the practice and experimental trials, and no scene configuration in the practice trials appeared in the experimental trials in either presentation list. There were two intransitive filler sentences, two transitive filler sentences, and one pronoun sentence in each practice session.

#### Procedure.

The experiment followed the same general procedure as Experiments 1–2 with a modified introductory sequence. As in the prior experiments, the sequence first introduced participants to the three types of aliens. Participants were then introduced to both action types (mimming, spinning). Participants then practiced a simple version of the task, describing scenes containing the minimum required number of aliens for the displayed actions. Participants described two mimming scenes (e.g., *the pinkies mimmed the bluey*) and two spinning scenes (e.g., *the greenies spun*). Participants were then shown a mimming scene with two groups of aliens and were told that in this context, a response in the form *the pinkies mimmed the bluey* does not provide enough information to identify which alien was mimmed (i.e., because there is more than one bluey on screen). Participants were told that a better description would be *the pinkies mimmed the bluey next to the greeny*. Next, participants were shown a spinning scene with two groups of aliens and were told that in this context, the response *the greeny spun* does not provide enough information to identify which alien spun (i.e., because there is more than one greeny), so a better description would be *the greeny next to the blueys spun*. Participants then moved onto the practice sessions. They were asked to describe for each scene who mimmed whom or who spun and to provide more detail in their responses by using words like *next to*.

Participants completed two practice sessions (5 trials each). The practice sessions followed the same format as in Experiments 1–2; the first practice was untimed, and the second practice was timed. During the practice, participants were shown the target sentence after giving their response. There was one target pronoun sentence elicited in each practice session. In the first practice session, the target pronoun sentence included a singular pronoun. In the second practice session, the target pronoun sentence included a plural pronoun. After completing the practice sessions, participants were presented with 74 experimental trials.

Crucially, the introduction to the task prior to the practice sessions included no mention of pronouns or example sentences using pronouns, and the experiment instructions never explicitly told participants to use pronouns in their responses. Participants saw only two examples of pronoun use before starting the experimental trials, and no particular attention was drawn to the pronouns in these examples. Furthermore, on the majority of trials (2/3), there was no need to use a pronoun in the scene description to avoid repeated reference. Consequently, the decision to use a pronoun in a response was likely to be driven by the participant’s own sentence planning decisions.

#### Data Processing and Analyses.

We restricted our analysis to the trials that elicited pronoun sentences. The data processing procedure and analyses of these trials followed the same format as in Experiments 1–2. One participant consistently used a preposition in their responses after the verb *mimmed* (e.g., *the greeny mimmed at the bluey next to it*). The use of *mimmed* as a phrasal verb was not considered to be an omission or disfluency error in the error distribution analysis, however this participant was excluded from the pronoun timing analysis in order to maintain consistency in sentence structure across all the trials in the analysis.

### Results

Of the 770 complete responses elicited in the pronoun trials, 760 of them contained pronouns (99%). Of the 792 pronoun total trial responses collected in Experiment 3, 61 responses were omitted from the analysis^10^ (8%). Of the remaining 731 responses, 53 contained disfluency errors (7%). See Supplementary Materials for a break-down of the omission and disfluency error types by condition.

#### Error Distribution Analysis.

**Figure 8.**
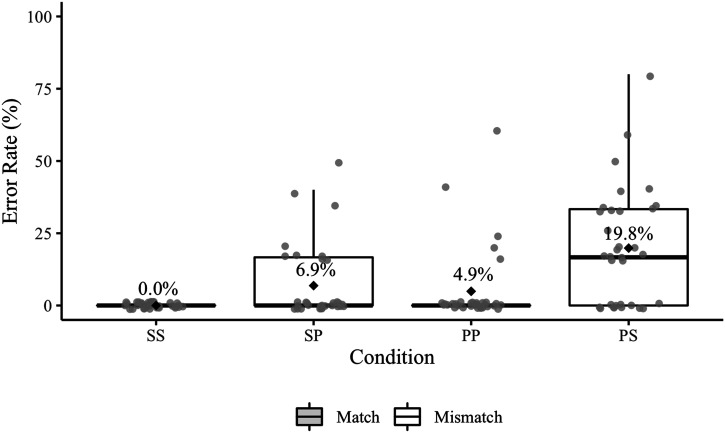
Experiment 3 participant agreement error rates. Mean error rates for each condition are labelled and identified by black diamonds. The grey points represent participant rates. The relevant condition comparisons (SS–SP, PP–PS) are positioned next to each other on the *x*-axis.

Experiment 3 elicited a total of 57 agreement errors (Table 11); 7 of these errors occurred in sentences that also contained a disfluency error. Figure 8 presents participant error rates by condition (including responses with disfluency errors). Recall that the relevant condition comparisons for Experiment 3 are SS vs. SP and PP vs. PS.

**Table 11.** Experiment 3 agreement errors and response distributions in the pronoun trials (values omitting responses with disfluency errors are given in parentheses).

Condition	Match Condition	Error Count	Response Count	Error Rate
SS	match	0	184 (173)	0%
SP	mismatch	13 (12)	186 (171)	7.0 (7.0)%
PP	match	8 (7)	178 (168)	4.5 (4.2)%
PS	mismatch	36 (31)	183 (166)	19.7 (18.7)%

In Experiment 3, the condition labels reflect the number of the antecedent + the attractor. The relevant comparisons are SS–SP and PP–PS, when the antecedent has the same number.

The posterior median for the overall effect of match was 1.99 (95% CrI [0.86, 3.53]), indicating that errors were more likely in the mismatch conditions. The posterior median for the overall effect of antecedent number was 1.45 (95% CrI [0.51, 2.89]), indicating that errors were more likely in conditions with plural antecedents. The posterior median of the interaction was −0.82 (95% CrI [−2.23, 0.12]), suggesting that there was not a reliable difference in the mismatch effect between conditions with singular and plural antecedents. The estimated probabilities of agreement errors were 0.0% (95% CrI [0.0, 0.6]) in the SS condition, 2.4% (95% CrI [0.3, 6.6]) in the SP condition, 6.0% (95% CrI [2.8, 10.8]) in the PP condition, and 18.1% (95% CrI [10.9, 26.6]) in the PS condition.

#### Pronoun Timing Analysis.

**Figure 9.**
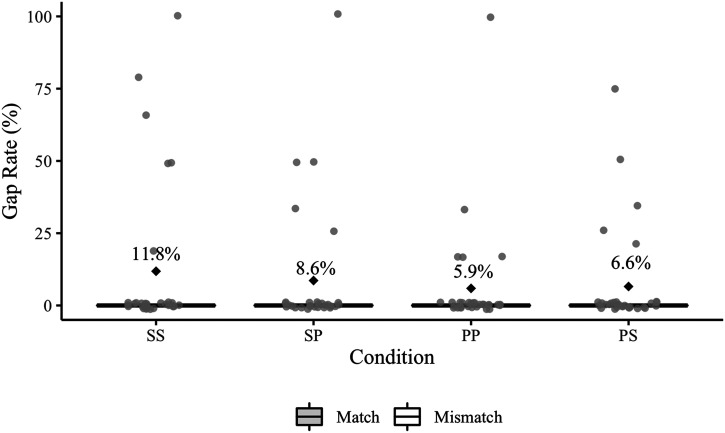
Experiment 3 participant pre-pronoun gap rates. Mean error rates for each condition are labelled and identified by black diamonds. The grey points represent participant rates. The relevant condition comparisons (SS–SP, PP–PS) are positioned next to each other on the *x*-axis.

The pronoun timing analysis was performed on 585 responses containing no agreement or disfluency errors. Two participants were omitted from the pronoun timing analysis: one because their responses could not be aligned, and one because they used a phrasal verb (see Data Processing and Analyses). There were 46 pre-pronoun gaps in the aligned responses (Table 12; see Figure 9 for participant gap rates). A plot showing the production time-course of the responses in the match and mismatch conditions broken down by sentence region is available in the Supplementary Materials.

**Table 12.** Experiment 3 pre-pronoun gap and response distributions.

Condition	Match Condition	Gap Count	Response Count	Gap Rate
SS	match	17	161	10.6%
SP	mismatch	11	149	7.4%
PP	match	11	149	7.4%
PS	mismatch	7	126	5.6%

In Experiment 3, the condition labels reflect the number of the antecedent + the attractor. The relevant comparisons are SS–SP and PP–PS, when the antecedent has the same number.

There were no reliable effects in the gap likelihood analysis. The posterior median for the overall effect of match was 0.26 (95% CrI [−0.71, 1.36]), the posterior median for the overall effect of antecedent number was −0.40 (95% CrI [−0.99, 0.19]), and the posterior median for their interaction was 0.09 (95% CrI [−0.51, 0.67]). The estimated probabilities of pre-pronoun gaps were 1.0% (95% CrI [0.1, 5.8]) in the SS condition, 1.5% (95% CrI [0.2, 6.6]) in the SP condition, 0.4% (95% CrI [0.0, 2.6]) in the PP condition, and 0.8% (95% CrI [0.1, 4.3]) in the PS condition.

### Experiment 3 Summary

The goal of Experiment 3 was to test whether pronoun number attraction errors arise in a context that engages the same reference decision processes as natural speech. In contrast to prior work (including our own Experiments 1–2), participants were not prompted to produce pronouns on every trial of the experiment. Rather, on one third of trials, a context arose in which participants could optionally produce a pronoun to avoid NP repetition. In these trials, participants decided for themselves whether or not to pronominalize in addition to deciding what form the pronoun should take. We found that our pronoun-inducing sentence context was highly effective at eliciting pronouns, with 99% of complete responses in these contexts containing a pronoun.

The distribution of pronoun number errors in Experiment 3 followed the same pattern as those in Experiments 1–2: We again observed a reliable pronoun number attraction effect, with errors more likely in the mismatch conditions than the match conditions (there were pronoun errors in 2% of match trials and 13% of mismatch trials). As in prior experiments, there was no reliable interaction between match and antecedent number, suggesting that the mismatch effect does not differ between the SS vs. SP and PP vs. PS comparisons.^11^ We thus see that the pronoun number attraction error effect persists even when there is more variability in the sentences participants produce (critically, pronoun-inducing contexts do not arise in all trials) and when participants decide for themselves whether or not to pronominalize, more similar to natural speech production.

In the Experiment 2 Summary, we suggested that eliciting pronouns in all trials and removing the decision to pronominalize may artificially inflate number attraction error rates through interference between trials and/or by changing the way that speakers access the message-level representation of the referent when planning pronoun form. We saw no evidence that the pronoun number attraction error effect was diminished in Experiment 3 when alleviating these concerns. In fact, a post-hoc comparison between the results of Experiment 3 and Experiment 2 (which elicited a similar target sentence construction) suggests that the error effect may even have been slightly larger in Experiment 3 (match × experiment posterior median −0.72, 95% CrI [−1.84, −0.003]; see Supplementary Materials). Interestingly, the observed error rates are more similar to those observed in prior pronoun experiments (Table 1) and in verb agreement attraction experiments (see Eberhard et al., 2005 for review).

Although we observed clear evidence of number attraction in the error distribution analysis, we did not see an analogous effect of match condition in the pronoun timing analysis. In fact, there were no reliable effects at all in the timing analysis. It is surprising that we do not even observe an antecedent number effect, despite the fact that this effect arose in both the error distribution and pronoun timing analyses in Experiments 1–2, and we observe the analogous effect in the Experiment 3 error distribution analysis (with errors more likely when the antecedent was plural). While it is possible that the lack of observed timing effects results from the increased variability in the sentences participants produce in Experiment 3, we believe that the analysis may have been underpowered to detect effects given that it only included 585 responses (the parallel analyses for Experiments 1–2 contained over four times as many responses). Therefore, the results of the pronoun timing analysis should be treated with caution.

## GENERAL DISCUSSION

The goal of the present study was to investigate how speakers plan pronoun form by testing whether/when this process goes awry. A better understanding of the processes involved in planning pronoun form will help to fill a gap in existing models of reference production, which often focus on the process of deciding to pronominalize but do not specify mechanisms for selecting pronoun form once this decision has been made (Arnold & Zerkle, 2019). In addition, an understanding of the processes involved in antecedent–pronoun agreement can be used to identify similarities and differences in the mechanisms used to establish the various forms of long-distance dependencies that appear in language. Across three experiments, we observed small but reliable pronoun number attraction effects, with speakers producing pronoun number errors in the presence of a nearby linguistic representation that differed in number from the pronoun’s antecedent. These effects were elicited using a scene description paradigm that more closely mimics natural speech (involving meaning-to-form mapping from a speaker-generated message) than the preamble paradigm previously used to elicit pronoun number attraction (Bock et al., 1999, 2004, 2006) (though see Accounting for Differences From Prior Experiments for some ways the present experiments were less natural). The pronoun number attraction effect persisted across different sentence constructions and when speakers engaged both the process of reference form selection (i.e., deciding whether to produce a pronoun) as well as that of pronoun form determination.

The presence of systematic interference from representations other than that of the pronoun agreement controller suggests that the correct pronoun form is not exclusively achieved as a by-product of accessing the message-level representation of the referent when deciding how to refer to it—an assumption implicit in many models of reference form selection (see Arnold & Zerkle, 2019). The fact that pronouns demonstrate susceptibility to agreement attraction effects suggests that their form is determined via a feature matching operation with the antecedent, potentially similar to subject–verb agreement. However, in contrast to prior experiments eliciting comparable number attraction rates between pronouns and verbs (Bock et al., 1999, 2004, 2006), the pronoun number attraction rates we elicited were smaller than those observed for verbs using a similar elicitation paradigm (Kandel et al., 2022). This contrast is likely due (at least in part) to the more naturalistic elicitation paradigm used in the present study (see Accounting for Differences From Prior Experiments). Furthermore, number attraction errors occurred even when the notional number of the pronoun referent was unambiguous and the agreement controller was a simple, unmodified noun (meaning that its number value should have been obvious to the speaker), presenting difficulty for Eberhard et al.’s (2005) extension of the marking-and-morphing proposal to antecedent–pronoun agreement.

In addition to investigating the presence of pronoun number attraction errors, we also looked for evidence of attraction effects in speech timing. In particular, we tested whether slowdowns prior to pronoun articulation were more likely in the same contexts when errors are more likely to occur—i.e., in the presence of an attractor with a mismatching number feature. Similar analyses with verbs have suggested that the same process or pressures that lead to attraction errors are active on more than just the trials in which overt errors are produced (see Kandel et al., 2022 for discussion). The results of the present study show preliminary evidence that the process or pressures that lead to pronoun number attraction errors may similarly be active more frequently than just the cases when errors occur, at least in the case of intervening attraction: In Experiment 2, speakers were more likely to pause before producing the pronoun in the mismatch condition than the match condition. While these pauses likely reflect additional attraction influences not captured in the error analysis, they were only produced on a small subset of mismatch trials, suggesting that even if overt errors underestimate the prevalence of the number attraction effect, this effect is still small.^12^ Furthermore, this timing evidence should be interpreted with caution, as we did not observe pronoun timing effects in Experiment 3, which elicited a similar target sentence structure (though this analysis was likely underpowered), or with non-intervening attractors in Experiment 1 (though note that a less homogeneous timing effect has been observed with non-intervening attractors for verbs; Staub, 2010). It thus appears that the pronoun number attraction effect in our study, while reliable, is small. Although a low incidence of pronoun number attraction contrasts with prior experiments showing pronoun number attraction rates comparable to verb attraction (Bock et al., 1999, 2004, 2006), it aligns with the lack of obvious pronoun attraction effects in natural speech (see Introduction).

In the remainder of the General Discussion, we discuss potential hypotheses for how speakers determine pronoun form (including directions for future research) as well as the source of the contrast between the number attraction effect observed in the present study and those elicited by prior experiments.

### Pronoun Form Determination

We identify two potential routes to pronoun form determination: (i) a conceptual route, in which speakers determine the necessary features for pronoun planning from the message-level representation of the referent (encompassing both Meyer & Bock’s, 1999 conceptual hypothesis and Schmitt et al.’s, 1999 pronoun access model), and (ii) a linguistic route, in which speakers determine the necessary features from the linguistic representation of the antecedent introduced in the prior discourse (encompassing broadly both the lexical hypothesis and tag hypothesis introduced by Meyer & Bock, 1999; this route can be instantiated via Eberhard et al.’s, 2005 marking-and-morphing hypothesis). These routes make different predictions about the susceptibility of pronouns to agreement attraction effects.

#### Conceptual Route to Pronoun Features.

The conceptual route predicts that pronoun form selection should be robust to interference from potential attractors, as speakers are able to access the necessary features for pronoun determination directly from the message-level representation of the referent that is (presumably) accessed during the process of reference form selection. Speakers may be able to access the relevant features for pronoun form directly from the message-level representation of the referent when these features are salient in the referent’s conceptual representation (e.g., notional number). This amounts to the strong form of the conceptual hypothesis introduced by Meyer and Bock (1999). However, the message-level representation of the referent cannot provide access to all features relevant for pronoun agreement cross-linguistically, such as grammatical gender. To account for this, Schmitt et al. (1999) proposed that access to the message-level representation of the referent provides direct access to the necessary features for pronoun form via mediation of a lemma representation.

The conceptual route to pronoun form may be necessary in contexts when pronouns do not have an established linguistic antecedent appearing in the prior discourse (e.g., if a speaker points to an individual on the street and exclaims, “He is tall!”). However, even when there is an available linguistic antecedent, the message-level representation of the referent is likely accessed during the decision to pronominalize, meaning that the conceptual route may still be readily applied—in these cases, the pronoun and linguistic antecedent would share features because they both encode the same message-level concept.

Under both the direct and mediated paths between the message-level representation of the referent and the pronoun features, as long as the speaker accesses the correct message-level representation of the antecedent, the correct pronoun form should follow. Thus, the conceptual route does not readily account for the agreement attraction error effect we observed in our study. To account for errors under the conceptual route, one must assume either (i) that speakers activate the wrong representation at the message level due to confusion (unlikely in our experiments since speakers were presented with a clear visual representation of the event), (ii) that the conceptual features of other message-level representations (e.g., a multiplicity feature) can cause interference (see Slevc et al., 2007 for attraction interference from conceptual gender), or (iii) that the links between relevant representations (e.g., in Schmitt et al., 1999’s model) are not in fact direct but rather are susceptible to interference—a revision that contradicts the primary assumption of the conceptual route (a direct path from the concept to the features). We propose that agreement attraction effects arise more naturally from the linguistic route to pronoun form planning.

#### Linguistic Route to Pronoun Features.

In the linguistic route to pronoun form, a speaker references information from the prior discourse to determine the pronoun’s features. More specifically, the pronoun features are determined via a matching operation with a linguistic representation of the antecedent. This representation has been proposed to take the form of a lemma (as in Meyer & Bock’s, 1999 lexical hypothesis), a trace of the phonologically-encoded form of the antecedent (as in Meyer & Bock’s, 1999 tag hypothesis), or a syntactic node (as in Eberhard et al., 2005).

Referencing how a referent has been previously encoded in the discourse is important for selecting the correct pronoun form when there is more than one possible way to refer to the referent. For instance, in English, the concept SCISSORS may be referred to using the plural NP *scissors* (corresponding to the pronoun *they*/*them*) or the singular NP *pair of scissors* (which can optionally be referred to using the pronoun *it*). Another motivation for the linguistic route is that in intra-sentential contexts, speakers independently need to monitor the content of the discourse as they decide how to refer to an entity in order to abide by sentence formulation constraints such as binding constraints or the avoidance of NP repetition. A linguistic route to pronoun features may thus arise as a by-product of this monitoring.

A consequence of relying on a linguistic representation of the antecedent to access the pronoun features is that having to refer to a prior discourse representation opens the door to interference from non-antecedent representations. The linguistic route thus can be used to explain the pronoun attraction effect observed in our study.

As mentioned in the Introduction, Eberhard et al. (2005) propose one mechanism of how attraction interference can occur in the linguistic route, inspired by marking-and-morphing accounts of agreement attraction in subject–verb agreement. In this framework, pronouns reconcile their number derived from agreement concord with a representation of the antecedent’s number value.^13^ The resulting pronoun number value is represented on a continuum from singular to plural (rather than a binary singular or plural distinction), and errors arise when this value falls further from the endpoints of the continuum, meaning the pronoun number value is less clear to the speaker. Crucially, Eberhard et al.’s (2005) hypothesis predicts that the likelihood of errors is influenced by the clarity of the antecedent number feature, in conjunction with the notional plurality of the referent. However, in our study, pronoun attraction effects arose even when the referent’s notional number was unambiguous and the subject phrase antecedent only contained a single NP (Experiments 2–3), meaning that the pronoun number value should have been obvious to the speaker. Consequently, our results present difficulty for Eberhard et al.’s (2005) instantiation of the marking-and-morphing proposal, which does not allow for the number value of the attractor to influence the reconciled antecedent number value (and thus pronoun number value) unless it is bound to the antecedent phrase. In order to account for errors in our sentences, Eberhard et al.’s (2005) model would have to allow the reconciled antecedent number value to be influenced by the number information of nearby morphemes that are not bound to it. In this case, marking-and-morphing could predict weak attraction in our sentences (similar to what we observe) due to the structural distance between the antecedent and the attractor.

In sum, while our results support a linguistic route to pronoun form, they are not explained by Eberhard et al.’s (2005) marking-and-morphing implementation of this route. In the next section, we propose an alternative account.

#### Accounting for Pronoun Number Attraction Errors.

Given the above, we propose that the agreement attraction errors observed in our experiments result from the implementation of a linguistic route to pronoun form determination. In this section, we suggest a potential mechanism for how these errors can arise within a linguistic route. This proposal is inspired by Schmitt et al.’s (1999) model of pronoun access and similarly attempts to provide a unified account of both reference form selection and pronoun form determination.

We adopt the same reference form selection mechanism as Schmitt et al. (1999), in which an *in focus* status of the message-level representation of the referent cues a speaker to produce a pronoun instead of a full NP. We additionally suggest that, as a speaker produces an utterance, they keep track of items active in the sentence representation to make sure they comply with formulation constraints (this monitoring, for instance, is what leads speakers to choose to pronominalize in the Experiment 3 pronoun trials). In our proposed model of pronoun formulation, when a speaker accesses the message-level representation of the referent, its *in focus* status prompts them to pronominalize and engage a retrieval process to look for the corresponding linguistic representation of the antecedent that is active in memory in order to access its features to determine pronoun form (similar to retrieval processes proposed for subject–verb agreement; e.g., Badecker & Kuminiak, 2007; Dillon et al., 2013; Wagers et al., 2009; inter alia)—it is in this process that errors occur. This retrieval process (based on theories of cue-based memory retrieval in content-addressable memory systems; McElree, 2000; McElree et al., 2003) may target linguistic representations that have particularly prominent mental status (e.g., high activation in working memory or an *in focus* feature at the lexical level), as this prominence is likely to co-occur with an *in focus* status at the conceptual level. Under normal circumstances, this strategy would help the retrieval process efficiently pick out the correct representation to serve as the agreement controller, whose features would then guide pronoun form determination. Referencing how the relevant referent was previously referred to in the sentence is important for maintaining discourse continuity within the speaker’s utterance.

Within this model, agreement attraction errors occur when there is more than one representation in memory with similar features that match (or partially match) the retrieval cues used to access the antecedent representation, leading the retrieval process to erroneously pull out the features of this representation for agreement instead of those of the antecedent representation. This situation would be more likely to arise when there is more than one representation with prominent mental status at the point when the feature retrieval process is engaged. This may occur in our experiment sentence frames, when the representation of the attractor is active during pronoun planning. In the Experiment 1 frame (e.g., *the pinkies above the bluey_i_ mimmed it_i_*), the attractor (*pinkies*) may have prominent mental status during pronominalization due to having special status as the sentence subject and/or because it was recently activated to compute verb agreement. In the Experiment 2–3 sentence frames (e.g., *the pinky_i_ mimmed the blueys above it_i_*), the attractor (*blueys*) is individuated by the prepositional phrase modifier containing the object of the mimming action, meaning it may have more prominent mental status due to recency and/or the fact that it is being individuated. Thus, in both sentence frames, an agreement error would occur in the following way: The speaker refers to the message-level referent of the object of the mimming action (e.g., PINKY), an *in focus* status of this representation prompts pronominalization, the feature retrieval system searches for a corresponding active linguistic representation, the retrieval process erroneously accesses the attractor representation (e.g., *blueys*) due to its prominent mental status and overlap with retrieval cues, and then the number feature of this representation is used for pronoun agreement (leading to the pronoun *them* instead of *it*).

This model can be used to explain the attraction effects observed in our study without relying on or ambiguity of the pronoun number value (as in a marking-and-morphing approach). However, it is important to recall that the error effects elicited in our experiments were small (with speakers producing the correct forms on most trials), and the observed timing evidence does not provide clear support that the process leading to errors is active on many more trials than those in which errors occur. While it is possible that errors are uncommon because the retrieval mechanism used to pull out the correct features for agreement is robust to interference and/or because the attractor representation does not always have sufficient mental prominence to compete for retrieval, this low incidence of errors could also arise if speakers at times use different routes to pronoun form determination.

Given that the contexts in which speakers produce pronouns vary, speakers may have more than one route to pronoun form available to them. For instance, speakers might be required to use the conceptual route when there is no linguistic antecedent in the discourse but may apply the linguistic route in cases of intra-sentential pronominalization or if there is a linguistic antecedent that is sufficiently salient in the discourse. In our experiments, even though there is an available linguistic antecedent, speakers still had the option to use the conceptual route to pronoun form, as the number feature necessary for pronoun agreement was salient in the message-level representation of the referent. Indeed, speakers are sensitive to notional number when producing pronouns, leading them at times to produce plural pronouns that reference grammatically singular antecedents (e.g., for collectives such as *fleet*; Bock et al., 1999). If our speakers frequently used the conceptual route (e.g., because it is less computationally intensive than the linguistic route, given that the message-level representation of the referent is already accessed during reference form selection), this may have lowered the rate of agreement attraction, as the conceptual route to pronoun form is likely robust to errors (see above). Future research should address the extent to which both conceptual and linguistic routes are applied during pronoun planning and what contexts prompt the use of each route.

Note that our proposed model makes several specific predictions about pronoun attraction errors that can be addressed by future research. For instance, it predicts that pronoun features other than number (e.g., grammatical gender) should similarly show attraction effects and that attraction rates should be comparable across different types of features, as the attraction would result from the same mechanism—i.e., retrieval of the incorrect antecedent representation from working memory. In fact, the model predicts that attraction errors of different features should co-occur (e.g., if the erroneously-retrieved attractor is plural and feminine, the pronoun should display both of these features), assuming that retrieval of the antecedent for agreement does not occur separately for each feature. The model additionally predicts fewer errors when potential attractors are not as prominent or *in focus* during pronoun planning; if the attractor is less prominent, it is less likely to be erroneously retrieved for agreement. Finally, our proposal suggests that sentence representations remain active and are thus available for reference during pronoun planning due to monitoring of sentence formulation constraints and that the use of the linguistic route may be applied intra-sententially to ensure maintenance of reference continuity. Future research should address whether attraction errors also arise in cases of inter-sentential pronominalization or whether the circumstances that give rise to the effect are exclusive to intra-sentential pronominalization.

### Accounting for Differences From Prior Experiments

A notable outcome of the present study is the difference in the shape of the elicited pronoun number attraction effect compared to prior experiments by Bock and colleagues (Bock et al., 1999, 2004, 2006; see Table 1 in the Introduction). For ease of comparison, Table 13 summarizes the number attraction error rates elicited in Experiments 1–3 and in Bock et al.’s (1999) tag pronoun condition (this data pattern is representative of the experiments presented in Table 1). This table shows several differences in the distribution of errors elicited in the present study and in Bock et al. (1999). For instance, while we observed an antecedent number effect in all three of our experiments, leading to more errors for plural antecedents than singular antecedents, Bock et al. (1999) elicited almost no erroneous uses of *it*. Moreover, and most importantly for the present discussion, the distribution of agreement attraction errors differs between the present study and that of Bock et al. (1999). We highlight two key differences below.

**Table 13.** Number error rates from Experiments 1–3 and Bock et al.’s (1999) tag pronoun condition.

Antecedent N	Attractor N	Exp 1.	Exp. 2	Exp. 3	Bock et al. (1999)
Singular	Singular	0%	1%	0%	4%
	Plural	3%	6%	7%	18%
	Mismatch − Match	3%	5%	7%	14%
Plural	Plural	3%	4%	5%	1%
	Singular	7%	11%	20%	2%
	Mismatch − Match	4%	8%	15%	1%
Match (collapsed)		2%	3%	2%	2–3%
Mismatch (collapsed)		4%	8%	13%	8–9%
Mismatch − Match		2%	5%	11%	6%

*Note*. This table does not use the same sentence condition labels as elsewhere in the paper (SS, SP, PP, PS), as the relevant pairwise contrasts were different for Experiment 1; the present labelling system allows for comparison across experiments. The ‘Match (collapsed)’ and ‘Mismatch (collapsed)’ labels are used to identify the match and mismatch error rates collapsing across both singular and plural antecedent conditions. As Bock et al. (1999) only report rounded error proportions and valid response counts for their four sentence conditions (no error counts), we calculated the maximum and minimum number of errors that could have produced the reported proportions and report the resulting maximum and minimum proportion of errors for the ‘Match (collapsed)’ and ‘Mismatch (collapsed)’ values. In Experiment 2, Experiment 3, and Bock et al. (1999), the attractor intervened between the agreement controller (the subject head) and the agreement target. In Experiment 1, the attractor did not intervene between the agreement controller and pronoun. Example Experiment 1 sentence: *The bluey above the greeny_i_ mimmed it_i_*. Example Experiment 2 sentence: *The bluey_i_ mimmed the greeny above it_i_*. Example Experiment 3 sentence: *The bluey_i_ mimmed the greeny next to it_i_*. Example Bock et al. (1999) sentence: *The actor_i_ in the soap opera rehearsed, didn’t he_i_?*.

First, Bock et al. (1999) observed a strong markedness asymmetry in the pronoun number attraction effect, with reliable attraction interference from plural attractors in the singular antecedent condition but virtually no interference from singular attractors in the plural antecedent condition. In contrast, in Experiments 1–3, we elicited attraction errors from both plural and singular attractors, and there was no reliable difference in the size of the agreement attraction effect based on attractor number (the match × antecedent number interaction was not reliable in any of the three experiments). While the increased error rates for sentences with plural antecedents in the present study may have in part reflected the overall main effect of antecedent number (likely resulting from an *it* bias), this increased difficulty for plural pronoun forms alone cannot explain the observed attraction effect (see Experiment 1 Summary).

We propose that the markedness asymmetry contrast arises due to differences in the elicitation paradigms used in the present study and in prior work. Recall that Bock and colleagues elicited sentences using a preamble completion paradigm, in which participants heard a sentence preamble that they were instructed to repeat and complete with a tag construction (Bock et al., 1999, 2004, 2006). As discussed in the Introduction, this elicitation paradigm differs from natural speech, as it relies heavily on comprehension and memory processes in addition to language production, and the form of the elicited utterance is driven by the provided preamble and task instructions rather than a message generated by the speaker. The present study, on the other hand, elicited sentences using a scene description paradigm that required speakers to generate a message to describe the events of a scene and then map this meaning to a sentence form.

The task demands of the preamble paradigm may have led to an increased markedness asymmetry in Bock et al. (1999). In fact, the contrast we observe for pronoun number attraction resembles that identified by Kandel et al. (2022) for verb number attraction elicited in different paradigms: Although verbs often display a stark markedness asymmetry in preamble elicitation paradigms (e.g., Bock et al., 1999, 2006; Bock & Miller, 1991; Eberhard, 1993, 1997; Thornton & MacDonald, 2003; Vigliocco & Nicol, 1998; but cf. Franck et al., 2002), this asymmetry appears reduced or absent in description paradigms (e.g., Kandel et al., 2022; Nozari & Omaki, 2022; Veenstra et al., 2014). Kandel et al. (2022) describe two hypotheses that attribute an increased verb markedness asymmetry to properties of the preamble paradigm, both of which can be adapted for pronoun agreement.

One hypothesis (proposed by Ted Gibson; see Kandel et al., 2022 for detail) suggests that a stronger markedness asymmetry arises in preamble paradigms in part due to misinterpretations of the preamble that are more likely for the SP condition than the PS condition. In Bock and colleagues’ experiments, in order to produce an appropriate pronoun form, participants must first parse the provided sentence preamble. If the participant incorrectly interprets the number of the antecedent in this preamble, this could lead them to produce a pronoun of the wrong form (e.g., Bergen & Gibson, 2012; Ryskin et al., 2021 for verb attraction). For instance, in a noisy-channel framework in which comprehension is influenced by interlocutors’ expectations about the presence of noise in the linguistic signal and the intended message of an utterance (e.g., Gibson et al., 2013; Levy, 2008), it is possible that individuals misinterpret SP preambles as PP preambles (accidentally inferring the subject head to have an omitted plural marking), which would lead them to erroneously produce a plural pronoun. It has been proposed that misinterpretations of SP preambles as PP preambles are more likely than misinterpretations of PS preambles as SS preambles due to differences in the relative frequencies of insertions and deletions of the English plural suffix *-s* and in the relative distributions of singular-singular and plural-plural NP-PP sequences in spoken English (Ryskin et al., 2021). This asymmetry would thus correspond to an asymmetry in the prevalence of errors for agreement targets (pronouns, verbs) that reference these preambles. We would not expect a similar asymmetry in the present study, as the scene description paradigm does not require interpretation of any provided linguistic material.

An alternative hypothesis proposed by Kandel et al. (2022) for verbs posits that the markedness effect arises because the preamble paradigm encourages different planning procedures from natural speech. In particular, participants in preamble tasks may use a generate-and-check strategy in which they plan the agreement target (in this case, the pronoun) upon first encountering the relevant agreement controller in the preamble. This strategy may be especially likely for Bock et al.’s (1999) tag pronoun condition since participants produced the same form of sentence continuation in every trial (a tag question) and thus would know upon first hearing the sentence subject in the preamble that they will need to refer to it using a pronoun. The initially generated pronoun form may then later be checked against the full preamble (e.g., as the participant is preparing to produce the tag continuation). If this feature checking process operates similarly to that proposed to underlie the markedness asymmetry for verb attraction effect in comprehension (e.g., Wagers et al., 2009), we may expect to see a similar asymmetry for pronouns in preamble elicitation paradigms. We believe that this planning strategy is less likely to be applied in the present study. Given the task design, participants are unlikely to plan the form of a pronoun before they know the rest of the sentence—particularly in Experiment 3, when participants do not produce pronouns on every trial and thus might not even know at the start of the sentence that they will want to produce a pronoun. Furthermore, to the extent that we observe attraction timing effects, they occur shortly before the pronoun, supporting the hypothesis that participants do not plan the pronoun well in advance of articulation.

The second key difference between the present study and those of Bock and colleagues is the size of the number agreement attraction effect when it occurs. In particular, in the singular antecedent conditions (when Bock et al., 1999 observed an attraction effect), the elicited agreement attraction error rates (mismatch − match) were much lower for Experiments 1–3 than for Bock et al. (1999) (Table 13). Note that there is precedent for attraction effects to be diminished in scene description tasks compared to preamble tasks; Kandel and Phillips (2022) found that the reflexive attraction effect observed in a preamble task disappeared when the same sentences were elicited in a scene description task.

We again propose that this contrast between our results and those of Bock et al. (1999) stems from differences across sentence elicitation tasks. For instance, the preamble paradigm used by Bock and colleagues to elicit tag pronouns may have biased speakers towards a linguistic route to pronoun form planning, which is more susceptible to error (see Pronoun Form Determination above). Since participants in preamble paradigms do not generate the messages of the utterances they produce, it is possible that they have a weaker representation of this message and thus cannot access the necessary features for pronoun planning from the conceptual level (as in the conceptual route to pronoun form planning). Furthermore, participants may be so focused on accurately repeating the preamble sentence before planning/producing their tag completion that they did not fully encode the message of this preamble and therefore have to reference the linguistic representation of the antecedent in memory (potentially in the form of a memory trace of the phonologically-encoded antecedent, as in Meyer & Bock’s, 1999 tag hypothesis). In contrast, in the present study, participants may be more likely to use a mix of the linguistic and conceptual routes to pronoun form (see Pronoun Form Determination), as the message-level representation of the referent should be available in the speaker-generated message. Using a mix of pronoun planning routes would lead to fewer errors, as the conceptual route is less susceptible to error.

Another factor that could have altered the likelihood of pronoun number attraction errors in the present study relative to Bock et al. (1999) is the elicited sentence construction. Our experiments elicited intra-sentential pronouns, whereas the pronouns elicited by Bock and colleagues appeared in a different clause from the main message of the utterance (e.g., in the tag *didn’t he*/*they*). It is possible that the participants in Bock et al. (1999) may have relied more heavily on the linguistic representation of the prior sentence to produce the pronoun in the sentence tag (similar to the hypothesis in the above paragraph), particularly since the message of the tag construction is reliant upon the prior clause. In addition, the pronoun antecedents in Bock et al. (1999) were complex subject phrases containing a subject head (the agreement controller) and a PP modifier containing the attractor noun. In contrast, in our experiments, the attractor noun was thus not part of the antecedent phrase. There is evidence that verb attraction effects are stronger from attractors that appear in a PP modifier of the subject than in more syntactically distant configurations such as relative clauses (e.g., Bock & Cutting, 1992)—thus, the syntactic position of the attractor relative to the agreement controller could have led to additional interference in Bock and colleagues’ sentence frames compared to those elicited in the present study.

It is possible that other differences between the elicitation paradigms could have influenced relative error rates, such as the fact that the experiments in the present study elicited sentences with a closed set of lexical items and syntactic constructions. All three experiments elicited responses with repeated referents, actions, and sentence frames, Experiments 1–2 contained no filler trials varying the type of event that occurred, and the event type was different (spinning vs. mimming) in only one third of Experiment 3 trials. In contrast, the sentences elicited by Bock and colleagues’ preamble experiments elicited sentences with unique referents and messages on each trial, including filler preambles on over half of the trials (Bock et al., 1999, 2004, 2006). This repetition of lexical items, syntactic constructions, and event types may have eased the burden of sentence planning for the speaker, resulting in fewer errors in the present study. There is evidence that sentence production is facilitated when speakers are exposed to message-level or sentence-level priming (Konopka & Kuchinsky, 2015), which may have occurred in our experiments when the same type of events were described using similar sentence frames across trials, and when planning can start earlier (Meyer et al., 2018), which may have been possible in our experiments when sentence frames and actions were repeated across trials. Indeed, the attraction error effect was smaller in Experiment 2 than Experiment 3 (which included filler trials), even though the elicited sentence constructions were similar in the two experiments, supporting the hypothesis that reduced variability in the task may lower error rates (though this difference is not on the same scale as the difference in error rates between our study and prior preamble studies).

Nevertheless, prior experiments have shown that verb attraction effects arise robustly even with lexically reduced item sets and no filler items (Kandel & Phillips, 2022; Kandel et al., 2022; Nozari & Omaki, 2022; Veenstra et al., 2014), which could suggest that the smaller attraction effect observed in the present study does not reduce to these properties. In addition, the variability in the sentences elicited by Bock and colleagues was contained within the part of the sentence provided to participants in the preamble; the part of the sentence that the speaker planned—the sentence tag containing the pronoun—was the same across all trials, including both filler and experimental trials (Bock et al., 1999, 2004, 2006). This tag construction involved a reduced lexical item set (*didn’t*, *he*, *she*, *they*), always used the same syntactic structure (*didn’t* + pronoun), expressed a similar message across trials (a request for confirmation of the preceding declarative event), and could begin to be planned as soon as participants heard the subject of the preamble. Thus, as in the present study, participants may have experienced syntactic and message-level priming of this construction between trials and had the potential to plan the construction early, thereby easing some of the planning burden and potentially lowering error rates. Consequently, the relative difference in response variability across paradigms is unlikely to be driving the relative difference in pronoun error rates.

In sum, while both the present study and those of Bock and colleagues elicited pronoun number attraction errors, the distribution of these errors may have been influenced by task-specific factors. These different distributions have consequences for the interpretation of how pronoun form is planned. For instance, an error distribution like that of Bock et al. (1999) that resembles the distribution of verb number attraction errors could be interpreted as indicating that similar mechanisms or representations underlie both subject–verb and antecedent–pronoun agreement (e.g., Eberhard et al., 2005). In contrast, the presence of different attraction profiles for verb and pronoun suggests that the underlying agreement processes are not uniform. Given the differences in task demands between the preamble elicitation paradigm used by Bock and colleagues (Bock et al., 1999, 2004, 2006) and the scene description paradigm used in the present study, we assume that the number attraction effect observed in the present study better reflects the pronoun attraction error rates that are likely to occur in natural speech.

It is important to note, however, that even though the scene description paradigm used in the present study engages key processes involved in natural speech (e.g., generating a message, form-to-meaning mapping of this message, etc.), there are still properties of this paradigm that make the task less natural and may have made participants more vulnerable to errors than in typical pronoun production. For instance, attraction interference may have been inflated due to the presence of a time limit, the use of novel words and concepts, the semantic similarity of the antecedent and attractor^14^, the visual salience of the attractor^15^, reduced/absent syntactic and semantic variability between sentences (especially in Experiments 1–2), and/or the lack of reference continuity across sentences. Despite this potential increase in attraction interference, the smaller attraction effect observed in the present study already helps to reconcile the discrepancy noted in the Introduction between the lack of obvious errors observed in natural speech and the high error rates previously elicited in the lab.

## CONCLUSION

The present study investigated how speakers determine the forms of the pronouns they produce. An understanding of this process can be used to inform more comprehensive models of reference production (which often lack explicit mechanisms for pronoun form selection) as well as to identify similarities and differences in the mechanisms used to establish different forms of long-distance dependencies (e.g., subject–verb agreement vs. antecedent–pronoun agreement). More specifically, we used number agreement attraction as a metric to determine whether the features for pronoun form are derived from the message-level representation of the referent accessed during the decision to pronominalize—either because they are salient in this representation (Meyer & Bock’s, 1999 conceptual hypothesis) or because this representation activates the corresponding lemma (Schmitt et al., 1999)—or whether the features are determined through an agreement operation with the antecedent, similar to subject–verb agreement. The former route to pronoun form (the conceptual route) predicts that pronouns should be robust to agreement attraction, as there is no opportunity for other representations to interfere. The latter route (the linguistic route), on the other hand, opens the door to interference from representations that appear in the pronoun’s linguistic context.

Across three experiments, we observed small but reliable pronoun number attraction effects, suggesting that pronoun form is (at least at times) determined by referencing the features of its linguistic antecedent. Sentences were elicited using a scene description paradigm involving meaning-to-form mapping from a speaker-generated message (in the style of Kandel & Phillips, 2022). The effects we observed persisted across different sentence constructions, when speakers engaged both the processes of reference form selection and pronoun form determination, and when the number value of the antecedent was clear and unambiguous (presenting difficulty marking-and-morphing accounts of antecedent–pronoun agreement; Eberhard et al., 2005). Interestingly, although we observed reliable error effects in all three studies, timing evidence for attraction (in the form of slowdowns prior to pronoun articulation) was weaker, contrary to the patterns previously observed for verb number attraction (Kandel et al., 2022).

We suggest that in situations of intra-sentential pronominalization, decisions about pronoun use and form may depend on other items in the sentence, rather than the message-level representation of the referent, because speakers must attend to these items to abide by sentence formulation constraints. We propose that speakers engage a retrieval process to reference the features of the linguistic antecedent and that this retrieval process can go awry if other representations in the pronoun’s linguistic context are highly prominent in working memory. Given that the participants in our experiments produced the correct pronoun form on the majority of trials, this retrieval process may be very robust to errors or speakers may be using a combination of conceptual and linguistic routes to determine pronoun form.

## ACKNOWLEDGMENTS

We would like to thank Claudia Pañeda for sharing her thoughts on pronoun formulation and Jeremy Zehr for his technical troubleshooting assistance. We are additionally grateful to the reviewers and action editor of this article for their helpful comments and to all of the individuals who participated in this research.

## FUNDING INFORMATION

This work was supported in part by NSF NRT grant DGE-1449815 to the University of Maryland (C. Phillips, PI).

## AUTHOR CONTRIBUTIONS

MK: Conceptualization, Data curation, Formal analysis, Investigation, Methodology, Project administration, Software, Visualization, Writing – original draft, Writing – review & editing. CRW: Conceptualization, Data curation, Investigation, Methodology, Software. CP: Conceptualization, Funding acquisition, Methodology, Supervision, Writing – original draft, Writing – review & editing.

## DATA AVAILABILITY STATEMENT

Data, analysis code, and supplementary materials are available from: https://osf.io/hgkjw/.

## Notes

^1^ In this paper, we use the term agreement to refer to feature matching between a target (e.g., a pronoun or verb) and a co-varying controller (e.g., an antecedent or subject). We do not commit to the claim that the same mechanisms underlie this matching across different linguistic dependencies (e.g., antecedent–pronoun or subject–verb dependencies).^2^ Though note that in Eberhard et al.’s (2005) model, pronoun number is additionally affected by the number marking on the root of the pronoun phrase and the lexical specification of the pronoun morpheme, derived from the referent’s notional number. This difference allows pronouns and verbs to express different number, even when the agreement controller is the same, thereby accounting for evidence that pronouns appear more sensitive to notional number than verbs when agreeing with collective nouns (e.g., Bock et al., 1999).^3^ These verbs were not used consistently in the participants’ responses.^4^ Note that this differs from Kandel and Phillips’ (2022) criteria, which omitted “’em” responses in order to more closely match the exclusion criteria used by Bock et al. (1999). Given the ubiquity of “’em” as a shortened form of “them”, we decided to allow this form in the present study.^5^ Note that in Experiment 1, the pronoun antecedent was part of the subject phrase (even though it was not the subject head itself), meaning that its number value may have been less obvious than in the Experiment 2 target sentence construction.^6^ Out of 96 trials (48 of which had plural target pronouns), one participant produced “it” on all trials, one participant produced “them” on only one trial, and one participant produced “them” on only three trials.^7^ In pairwise comparisons, both the SS–SP contrast (posterior median 1.99, 95% CrI [1.02, 3.05]) and the PP–PS contrast (posterior median 1.32, 95% CrI [0.56, 2.12]) were reliable. These comparisons were computed using the same models from the error distribution analysis with dummy coding.^8^ These participants never produced the pronoun “them” in a response. Two of these participants produced 24 pronouns total, two produced 23 pronouns total, and two produced 20 pronouns total.^9^ Two of these participants produced no pronouns in any of their responses.^10^ 10 of the 39 complete omitted responses (26%) did not include a pronoun.^11^ In pairwise comparisons, both the SS–SP contrast (posterior median 3.18, 95% CrI [1.23, 5.49]) and the PP–PS contrast (posterior median 2.15, 95% CrI [0.98, 3.66]) were reliable. These comparisons were computed using the same models from the error distribution analysis with dummy coding.^12^ Though it is possible that there was a smaller, more uniform timing effect leading to pre-pronoun slowdowns that the forced-aligner was not sensitive enough to detect.^13^ During agreement concord, pronouns get their features from the referent via the same process of satisfying semantic constraints that guides speakers to choose a specific noun during lexical selection (Bock et al., 2006; Eberhard et al., 2005).^14^ Shared features such as animacy and semantic relatedness may lead to additional interference retrieving items from memory (Gordon et al., 2001) and thus increase number attraction rates for verbs (Barker et al., 2001).^15^ Since the attractor was visible on the screen as participants produced their responses, it is possible that the visual representation of the attractor number (singular or multiple) could have caused interference. If one assumes that only the aliens in the target group (where the mimming action occurred) can produce this interference, this interference would lead to more pronoun number errors in the mismatch conditions than in the match conditions (differences between match conditions are less likely when considering the whole scene, as the vast majority of trials included at least one group of aliens of each possible number). However, if this visual interference were the sole cause of the agreement attraction interference elicited in the scene description paradigm, we would expect to see this interference in Kandel and Phillips’ (2022) reflexive trials in addition to their pronoun trials; even though both trial types were elicited with similar scenes, they only observed attraction errors in the pronoun trials.
